# Exploring the moderating role of maternal attachment style on the impact of a virtual reality environment on parental empathy and style: A pilot study

**DOI:** 10.1371/journal.pone.0355199

**Published:** 2026-08-24

**Authors:** Jac Neirin Airdrie, Domna Banakou, Crescent Jicol, Karin Petrini, Mel Slater, Catherine Hamilton-Giachritsis

**Affiliations:** 1 Department of Psychology, University of Bath, United Kingdom; 2 The Wolfson Centre for Young People’s Mental Health, Department of Psychological Medicine, Cardiff University, United Kingdom; 3 School of Psychology, Cardiff University, Wales, United Kingdom; 4 Event Lab, Department of Clinical Psychology and Psychobiology, University of Barcelona, Passeig de la Vall d’Hebron, Barcelona, Spain; 5 Arts et Métiers Institute of Technology, LAMPA, France; 6 Bath Institute for the Augmented Human (IAH), Department of Computer Science, University of Bath, Bath, United Kingdom; 7 The Centre for the Analysis of Motion, Entertainment Research and Applications (CAMERA), Bath, University of Bath, United Kingdom; 8 Institute of Neurosciences of the University of Barcelona, Barcelona, Spain; 9 Bath Institute for Digital Security and Behaviour, University of Bath, Bath, United Kingdom; Public Library of Science, UNITED KINGDOM OF GREAT BRITAIN AND NORTHERN IRELAND

## Abstract

Research has demonstrated that parental empathy, a factor implicated in risk of child maltreatment, could be enhanced using Virtual Reality (VR) by helping mothers to take a child perspective. However, whether parental attachment style could affect the efficacy of this approach is unclear. In this pilot study, a sample of nineteen British mothers participated in a VR study where they were embodied in a child avatar and interacted with a virtual mother adopting a harsh and critical (negative) or warm and supportive (positive) parenting style. Participants completed the Adult Adolescent Parenting Inventory, a measure of risk of child maltreatment, including an empathy subscale, before and after exposure to both conditions. They completed the Parenting Scale, a self-report measure of parenting style, and the Mind in the Eyes task (cognitive empathy) before and after exposure to each individual condition. Participants’ attachment style was measured using the Experiences in Close Relationships Scale. Mothers’ scores for empathy did not change from before to after the exposure, and the extent of change did not relate to attachment style. However, there was a significant increase in attitudes relating to effective parenting practices (lower laxness, overreactivity and verbosity), with some preliminary evidence this change was moderated by attachment style. Thematic Analysis of participants’ qualitative reports of the experience suggested the VR evoked cognitive and emotional responses congruent with each condition, led to child perspective-taking, meta-cognitive awareness of their communication styles, and self-reported changes in parenting behaviours. It also highlighted areas for further development of the VR paradigm to enhance immersion. The lack of replication of a change in empathy requires further investigation with more robustly validated measures of both cognitive and emotional empathy. Increases in self-reported effective parenting style, which appeared to be moderated by parental attachment style requires further investigation with a larger sample and better representation of the full distribution of attachment styles and socioeconomic, cultural and ethnic backgrounds. Whilst not replicating previous findings of changes in empathy, an increase in self-reported effective parenting style provides some preliminary support for the potential future utility of VR in reducing child maltreatment.

## The impact of maternal attachment style on the efficacy of a virtual reality environment for increasing parental empathy

Incidence of child maltreatment in the UK has been steadily increasing from 22.5 per 100,000 in 1997 to 60.1 per 100,000 in 2017 [1]. Children who are victims of such abuse have increased risk of mental, physical and behavioural difficulties and are more likely to both be a victim of, and perpetrate future violence (see McCrory et al., 2011 [2]for a review). A recent conservative estimate of the economic cost for society per each surviving victim of child maltreatment was £89,390, whilst the cost of a death resulting from maltreatment has been estimated to be £940,758 per child [3]. Research has highlighted a number of relevant risk factors for child maltreatment. For example, a meta-analysis of 155 studies, found a parent perceiving a child as a problem, parental anger and self-esteem, family conflict and cohesion were predictive, with a large effect size, of child physical abuse and neglect [4]. A factor associated with a number of the factors above [5–8], which itself has been implicated in risk of maltreatment, is the adult attachment style of the parent [9]. Attachment style is a model of how one views oneself, others, and relationships based on early experiences with caregivers [10]. Importantly, Bowlby [11] predicted a parent’s own attachment experiences would shape one’s quality of parental caregiving (caregiving behavioural system) possibly due to it influencing parents’ later ability to infer the mental and emotional states of themselves and others [12,13].

Broadly, previous research has adopted one of two approaches for determining an individual’s attachment as an adult. Interview-based measures (i.e., the Adult Attachment Interview; AAI) assess individuals’ narratives of their early experiences to understand their states of mind in regard to attachment behaviours [14]. An alternative approach to measuring adult attachment is via self-report measures [15]. Importantly, self-report measures of adult attachment may not represent well one’s early attachment experiences due to the potential for individuals, particularly of insecure attachment, to minimise the more difficult aspects of their relationship with a caregiver [16]. Therefore, self-report measures ask about individuals’ experiences of adult romantic relationships to define adult attachment, which may not always correspond closely to their early experience of relationships [17]. Consequently, the self-report approach may be best viewed as a measure of an individual’s current sense of safety and security within relationships.

When using the latter approach, adult attachment has been defined along two dimensions: avoidance and anxiety [18]. Individuals high in attachment avoidance are characterised by self-reliance, experience discomfort depending on others, and have a tendency to deactivate the attachment system to cope with distress. Conversely, individuals high in attachment anxiety desire closeness to others, fear rejection, and have a tendency to hyperactivate the attachment system to cope with distress [19]. Individuals low on both dimensions (secure) have a positive view of themselves and others, and a “capacity to rely trustingly on others when the occasion demands” [11].

The parental attachment system and caregiving behavioural system are thought to be mutually exclusive (as one becomes activated, the other becomes deactivated [20]). Therefore, if a parent’s attachment system becomes activated, their own needs and strategies (which differ by style) may interfere with an ability to respond appropriately to their child’s needs [21]. Notably, threat is thought to activate the attachment system [19] (and see [22] for experimental support of this). A child displaying distress signals could be perceived as threatening [23] and, therefore, activate the parent’s own attachment system and associated coping strategies, which could interfere with the parents’ capacity to engage in appropriate caregiving behaviours [19].

Jones et al. [24] conducted a review of studies exploring the relationship between attachment and parenting behaviour. Six studies found a relationship between adult attachment and child maltreatment such as increased rates of insecure attachment amongst maltreating parents (59%) [25] compared to the general population (42%) [26] and found that those with insecure attachment scored higher on measures of child abuse risk [27–29]. Further, a meta-analysis of 16 studies found parental insecure attachment was significantly related to child maltreatment (Odds Ratio of 2.93) [9]. However, rather than insecure parental attachment being a direct risk of maltreatment, it has been conceived that having a secure attachment style may serve as a buffer against other life stressors and experiences that would otherwise lead to an increase in risk [30]. It is possible that the buffer of a secure attachment style against maltreatment risk might be related to an individual’s capacity to empathise. Indeed, those with secure attachment styles have been found to have a higher level of empathy compared with those with insecure attachment styles [31,32]. In a related area of literature, low empathy was found to mediate the relationship between adult attachment and intimate partner violence [33]. Therefore, it is possible that increasing the capacity to perspective-take and empathise in individuals with insecure attachment may reduce risk of child maltreatment.

Immersive Virtual Reality (VR) has been argued to be effective in facilitating perspective-taking [34] and some forms of empathy [35,36]. This is achieved by placing a participant in the body of a virtual avatar (embodiment) which creates the illusion of body ownership whilst interacting with a virtual environment. Such techniques have demonstrated success in embodying adults in the body of a small child, leading to perceptual and attitudinal changes [37]. Hamilton-Giachritsis et al. [38] applied this technique in a group of 20 Spanish mothers not at high risk of maltreatment. Participants were embodied as a 4-year old child and interacted with a mother avatar that responded in a positive (warm and supportive) or negative (harsh and critical) manner. Participants reported a strong body ownership illusion and had cognitive, emotional, and physical responses consistent with this. They also found that participants’ levels of self-reported empathy increased following exposure to the negative mother. Therefore, it is possible that such a technique could be used in the future to help reduce risk of maltreatment in parents. However, given that parents at higher risk of maltreatment are more likely to have insecure attachment styles [25]and given that it is such parents that the virtual reality intervention we describe here is hoped to benefit, a key research question is whether attachment style might moderate any changes in empathy following exposure to the VR experience. It is possible that exposing individuals with insecure attachment to the VR environment may evoke a threat response and hence activate their own attachment needs [19] and therefore find it more difficult to take the perspective of a child during immersion, hence leading to smaller changes in empathy or increases in effective caregiving behaviours. On the other hand, it may be that the virtual nature of the environment, whilst evoking an emotional response, may not overwhelm them in the same way a similar situation in real life might do, and therefore provides a safe opportunity for perspective-taking. Hence, the primary aim of the present pilot study was to use the VR technique used by Hamilton-Giachritsis et al. [38] to explore whether (a) empathy in participants increased following virtual child embodiment and exposure to a negative and positive mother in VR and (b) this increase was moderated by participant attachment style. The secondary aim was to explore whether parents would self-report less dysfunctional parenting styles after exposure to the environment. Both a positive and negative virtual mother were used to remain consistent with Hamilton-Giachritsis et al. [38]. The study was predominantly interested in examining the effect of exposure to both conditions in combination on empathy and how this related to attachment style from before to after exposure. However, based on the previous study’s finding that empathy increased only after exposure to the negative mother, we also measured empathy (using a Mind in the Eyes task) and parenting styles after each condition separately. A third aim was to explore participants’ subjective experience of the virtual environment including participants’ cognitive, emotional and behavioural response to preliminarily explore any mechanisms and moderations of change. We hypothesised the following for this study:

1. Participants will report feeling embodied in the child avatar.
2. Participants’ empathy scores will be higher after exposure to the virtual mothers than before exposure and that this change will be larger following exposure to the negative mother [38].
3. Parents’ risk of maltreatment scores overall will be lower following exposure to the virtual mothers than before.
4. Participants will self-report more effective parenting strategies after exposure to the virtual mothers than before exposure and the change will be larger following exposure to the negative mother [38].
5. Attachment security will moderate the extent of change in empathy and self-reported parenting style such that higher scores in attachment security will be associated with larger increases in empathy and effective parenting styles following exposure to IVR.

## Method

### Participants

Participants were mothers over the age of 18 years with at least one child under the age of five years old. They were fluent in English with no known parenting difficulties. They were recruited between 23^rd^ May 2019 and 14^th^ Match 2020 from the local area via posters, email adverts and social media posts targeted at local institutions (i.e., nurseries, schools, universities, parent and child play groups, gyms, exercise classes, libraries and leisure centres). Participants were excluded if they had a diagnosis of epilepsy, significant back or neck difficulties, had recently consumed alcohol, or had contact with Children’s Services in relation to their childcare. Participants who reported susceptibility to motion sickness were included but warned of the potential for the VR environment to induce such symptoms. Nineteen mothers (sample characteristics are presented in Table 1) between the ages of 22 and 44 years took part (*M age* = 35.5, *SD* = 5.6). Most participants were white, married, from a high socioeconomic background, and had reached a high level of education. No participants reported any motion sickness whilst taking part. No participants reported prior abuse perpetrated by someone outside their family whilst three reported abuse perpetrated by someone inside their family. Ethical approval was obtained for this study from the University of Bath Psychology Research Ethics Committee (PREC code: 19–102). Participants provided their written informed consent to take part. As part of the debrief, participants were provided with contact details of local organisations specialised in supporting people who have experienced abuse.

**Table 1:** Sample Characteristics (N = 19).

Demographic variable	*Mean (SD)*
Age years	35.5 (5.6)
Age of youngest child	2.1 (0.9)
	*n (%)*
No. children	
One only	9 (47)
Two	10 (53)
White^a^	15 (79)
Married/long-term relationship	18 (95)
Highest level of education	
Postgraduate Degree	11 (58)
University Graduate	5 (26)
Currently at University	1 (5)
High School Graduate	2 (11)
Work status	
Full-time	6 (32)
Part-time	10 (52)
Full time education	1 (5)
Stay at home mother	2 (11)
Household income (£)	
< 15,000	1 (5)
15,001–25,000	3 (16)
25,0001–30,000	0 (0)
30,001–40,000	1 (5)
40,001–60,000	6 (32)
Over 60,000	7 (37)
Don’t know	1 (5)

*Notes.*

^a^of the four non-white participants three were Asian and one multi-racial, all were UK nationals.

### Design

A within-groups, counter-balanced design with a single binary factor ‘Mother Avatar Behaviour’ was used. Hamilton-Giachritsis et al. [38] found participants who were exposed to the negative mother before the positive experienced issues with wash-out such that they had difficulty trusting the positive mother and feared that, whilst being nice in the moment, she could at any time become critical again. Therefore, we were also interested in the impact of the order in which participants were exposed to the mother conditions. Participants were randomly allocated to one of the two designed groups: experiencing the positive mother first and then the negative mother (positive then negative), or the negative mother first and then the positive (negative then positive). The two sessions were separated by a week. Quantitative and qualitative data were collected to obtain a more complete picture of changes in empathy and self-reported parenting behaviours. Data were collected at four time points. Qualitative data were also used in an exploratory way to understand participants’ experience of the VR, explore possible mechanisms and of change and explore any barriers to inform future refinements to the paradigm (see Table 2 for details as to which measures were administered at each time point).

**Table 2:** Summary of study procedure.

	Time 1	Time 2 (one week later)
	*Pre VR*	*Pre VR*
1.	AAPI (Form A or B)	-
2.	Parenting scale	Parenting scale
3.	Mind in the eyes (counterbalanced A or B)	Mind in the eyes (counterbalanced A or B)
	*VR*	*VR*
4.	VR paradigm (positive or negative)	VR paradigm (alternative condition to that at Time 1)
	*Post VR*	*Post VR*
5.	Post experience questionnaire and Semi structure interview	Post experience questionnaire and Semi structure interview
6.	Parenting scale	Parenting scale
7.	Mind in the eyes (alternative version to that used at Pre)	Mind in the eyes (alternative version to that used at Pre)
8.	-	ECR-R
9.	-	AAPI (same version as Time 1)

### Procedure

Participants arrived at the VR lab at the Psychology Department at the University of Bath. They read an information sheet and subsequently had the opportunity to ask questions, they then completed a consent form. Participants were informed that taking part was voluntary and they could stop at any time without giving a reason. The order (as well as information on counterbalancing) that participants completed the measures of the study are summarised in Table 2.

#### VR paradigm.

**Fig 1:**
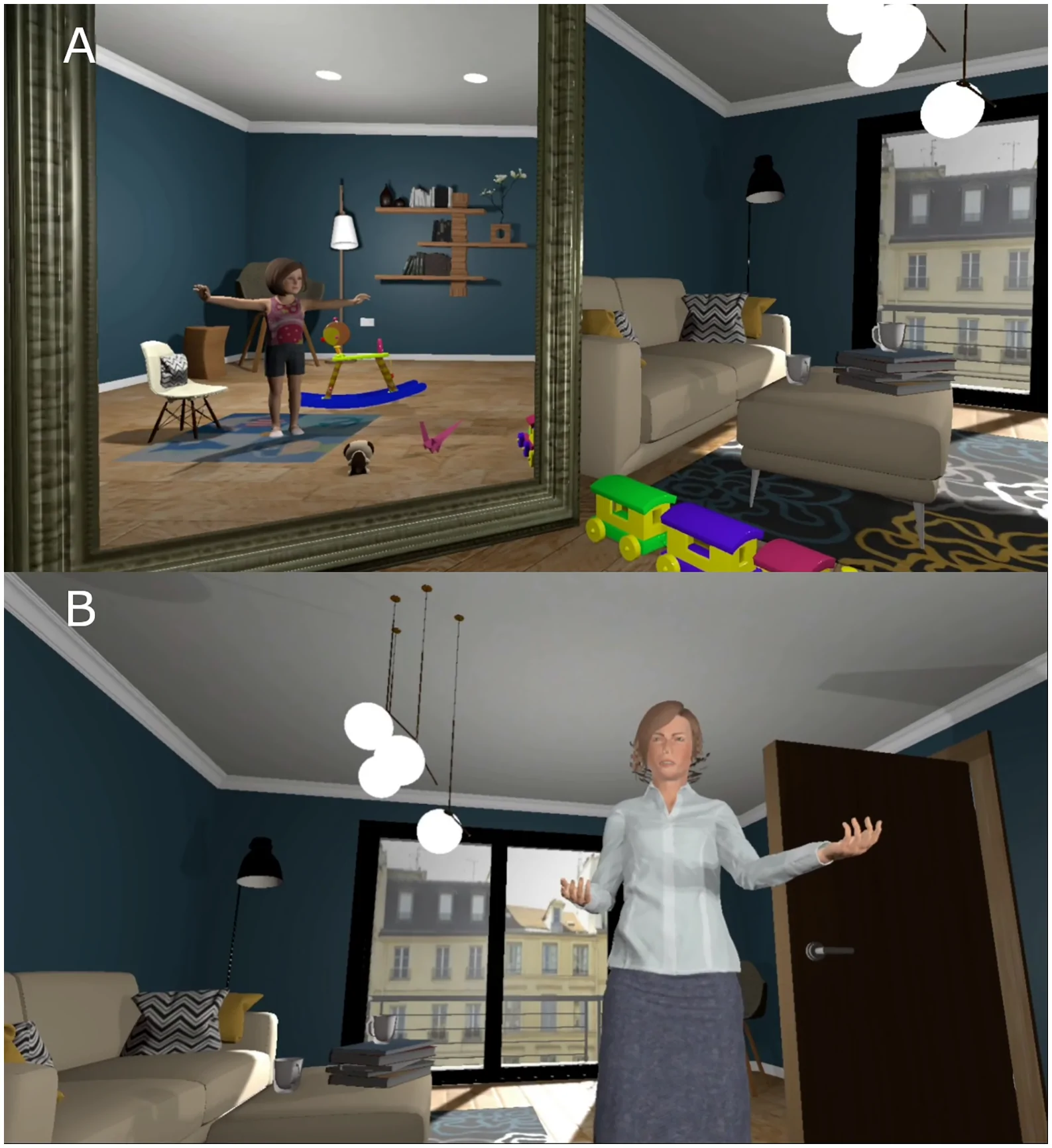
Virtual Reality Environment and Set-up (A) Participant’s body substituted by a child virtual body and visible in a virtual mirror. (B) Participant point of view of the mother avatar in the negative condition.

Having suited up with the VR equipment, participants entered the virtual living room (comprehensive detail about equipment and the virtual environment, including information on the child and mother avatars, the dialogue, the room itself (S1 File) and a video clip demonstrating the embodiment calibration procedure and interactions with the virtual mother (S2 File) can be found in Supplementary Information). Using pre-recorded instructions, participants were first asked to look around the room and describe verbally what they saw, they were subsequently asked to do some physical exercises whilst looking at their own virtual body in the mirror. Their virtual body was represented as a child of about five years old. They saw this body both by looking down towards themselves and in the virtual mirror. Their upper body movements were mapped to the virtual body in real-time, i.e., synchronously and correspondingly (see Fig 1). This lasted for around five minutes. The virtual mother then entered the room and interacted with the participant. The type of interaction varied according to whether it was the positive (PC) or negative (NC) condition. The Negative mother adopted an emotionally harsh tone, was critical and blaming of the child’s (i.e., participant’s) actions and labelled the participant as “naughty”. The positive mother, on the other hand, adopted an emotionally warm and soothing tone, gave the suggestion of engaging in shared play and asked about the participant’s preferences. She was also complimentary about the participant’s character and shared that she loved them. The interview and questionnaires then followed the VR condition. Participants returned a week later to complete the second condition and questionnaires. Having completed the study, participants were verbally debriefed about its aims and given opportunity to ask questions. They were given a debrief form which included sources of support if participants had experienced abuse or were concerned about their own parenting. Participants were reimbursed £10 for taking part.

### Measures

#### The Adult Adolescent Parenting Inventory version 2.1 (AAPI 2.1).

The AAPI is a self-report measure of parenting attitudes (Inappropriate Parental Expectations, Lack of Empathic Awareness, Values Corporal Punishment, Parent-Child Role Reversal and Oppressing Power and Independence) associated with increased maltreatment risk. There are two versions (Form A and B) each with 40 items. In the Hamilton-Giachritsis et al. [38] study Form A was used before and Form B after exposure to the VR conditions in line with measure developer guidance. Due to potential inconsistencies between the forms (see S11 File for a discussion of this), in the present study participants used either Form A or Form B at both time points. Participants responded on a five-point Likert scale ranging from one (strongly agree) to five (strongly disagree). The AAPI has shown to have good ability to discriminate between abusive and non-abusive parents (Cohen’s d between .68 and .98), and good internal reliability (Spearman Brown *r*s ≥ .82 [39]). The present study is mainly interested in Empathic Awareness, however, will explore all constructs as well as total risk in view of the total score being shown by other studies to be more reliable than the individual constructs [40]. The AAPI was administered before exposure to any mother conditions and after participants had been exposed to both conditions, therefore it was only possible to explore the combined effect of both conditions on change in this measure.

#### The Parenting Scale.

The Parenting Scale [41] is a 30-item measure of dysfunctional discipline practices. It consists of three factors: Laxness (parents give in, allow rules to go unforced, provide positive consequences of misbehaviour), Overreactivity (displays of anger, meanness, and irritability) and Verbosity (lengthy verbal responses and a reliance on talking even when talking is ineffective). Participants responded on a seven-point Likert scale ranging from a response reflecting a behaviour considered high in one of the factors (e.g., Overreactivity) at one end and low in the factor at the other end. Higher scores indicate less effective parenting strategies. The scale has been found to have acceptable reliability and validity [15]. Participants completed this scale before and after exposure to each condition and therefore we could explore the impact of each condition separately (as well as the combined effect). Before exposure to the first VR condition participants were asked about their parenting during the past two months and afterwards asked about how they thought they would parent in the future. When they returned a week later and before exposure to the second condition they were asked about their parenting during the past week and again after exposure to the second condition they were asked about how they will try to parent in the future.

#### Reading the mind in the eyes.

The Reading the Mind in the Eyes Test-Child Version (REMT; [42]) was used to measure cognitive empathy. The task involves describing the emotional state (4-option multiple choice) of a child based only on their eyes. Images were used from the online NIMH-chEFS Picture Set [43]. Participants completed this measure on four occasions, before and after the negative condition, and before and after the positive condition. Participants were presented with different versions (which comprised different faces) before and after taking part in each condition to help control for practice effects. A laptop with a 13.3-inch monitor was used to present the images using the Inquisit software (https://www.millisecond.com/).

#### Post experience embodiment questionnaire.

Participants’ virtual experience was assessed by a questionnaire after each condition in order to measure body ownership (Embodiment; see S3 File). It included two questions on body ownership (MyBody; Mirror), three on how they responded cognitively and emotionally to the mother, one on agency (Agency), four on whether they felt like a child (Younger; FeltChild; Childlike) and two control questions (Features; TwoBodies). Participants responded on a −3 to +3 Likert scale ranging from strongly disagree (−3) to strongly agree (+3).

#### Experiences in Close Relationships scale Revised (ECR-R).

Adult Attachment anxiety and avoidance was measured using the ECR-R [15]. Participants responded to a 36-item measure, using a seven-point Likert scale between one (strongly disagree) and seven (strongly agree). The ECR-R has demonstrated good internal reliability (Cronbach alphas > .93) and validity [44,45]. This was administered on a single occasion after participants had completed all other measures and conditions in order to reduce priming effects.

#### Semi-structured interview of participants’ experience.

The qualitative component involved a semi-structured interview (S4 File) of participants’ subjective experience of the VR environment immediately following each condition. Some questions lent themselves to a more inductive approach to analysis, i.e., “How was that experience?” whereas others were more deductive in that they asked specifically about participants’ “thoughts” and “feelings” in response to each mother condition as well as asking them if the experience will make them think about how they respond to their own children. The interview also asked participants if the experience would make them behave differently towards their children and to provide a justification for the answer they gave. After the second VR condition an additional question asked about whether the experience of the VR the previous week affected how they responded to their children in the subsequent week.

### Data analysis

#### Quantitative.

All analyses were conducted using IBM SPSS statistics version 25. Due to a small sample and where data was not found to meet the assumptions of parametric testing (normal distribution was checked using the Shapiro-Wilk statistic (S5 File), whilst homogeneity of variance was checked with a Levene’s test), non-parametric equivalents were used to check if this resulted in different findings for main effects (not possible with interactions) and correlations. Internal reliabilities of all questionnaires in the present study were checked with the Cronbach’s alpha statistic. One participant failed to complete half of an AAPI questionnaire so was excluded from this analysis. Analyses of difference in AAPI raw scores from before to after being exposed to the combination of both VR conditions (Time A vs Time D) was conducted using a factorial analysis of variance (ANOVA) with VR mother order (PC then NC vs NC then PC) as a between-subjects and Time (A and D) as a within-subjects factor. Similarly, analyses of change in mean score for the Parenting Scale and its subscales, and proportion of emotions identified correctly for the REMT (the counterbalanced order was also explored as a between subjects factor) before and after each condition (A vs B and C vs D) separately and for the combined effect of both conditions (A vs D), were conducted using a repeated measures ANOVA with VR mother order (PC then NC vs NC then PC) as the between-subjects factor and Time as within subjects factors. Participants’ responses to the post-experience questionnaire were checked to ensure responses were consistent with embodiment and differences between the PC and NC were compared with t-tests. Change scores were calculated for the key study variables. For the AAPI, participants’ raw scores overall and for each construct separately at Time D were subtracted from their corresponding scores at Time A, such that negative scores indicate a reduction in maltreatment risk. For the Parenting Scale, scores after the VR condition were subtracted from scores before the VR condition such that positive scores show a change towards more effective (less dysfunctional) parenting. For the REMT, pre-VR scores were subtracted from post-VR scores such that positive scores reflect higher percentage correct at post-VR compared to Pre-VR. The relationship between these change scores and attachment anxiety and avoidance was explored via simple correlations (due to not meeting sample size requirements to power regression).

#### Qualitative.

Participants’ responses to the semi-structured interview were analysed using Thematic Analysis following the guidance of Braun and Clarke (2006) [46], using an essentialist/realist epistemological stance and a combined inductive and deductive approach [46]. The first author (JA) listened to and transcribed all interviews, re-read all transcriptions for both the PC and NC and generated and applied semantic level codes to the data. The codes were then grouped together into themes and subthemes. The author sought to identify themes that were *semantic* in nature in that they represent explicit or surface level meanings in the data [46]. A second author (CH-G) reviewed the process of code generation and grouping into themes offering their perspective and interpretation which led to the further refinement of themes. Triangulation between quantitative and qualitative data took place at the inferential stage [47].

## Results

### Embodiment

Participant median scores (and interquartile range; IQR) for embodiment (see S6 File) were generally high with the interquartile range subsuming only positive responses for participants feeling like they were in the child’s body, feeling like the mother was acting like they were are child, feeling like a child, the mother being aware of their presence, and the avatar movements being caused by their own movements, which suggested successful embodiment. However, for two crucial questions of “I felt like the body was my own body” and “I felt like I was looking at myself in the mirror”, whilst the overall median was in the positive direction, the IQR included negative response options of the scale suggesting that for some participants, successful embodiment may not have taken place. Examination of participants responses indicated that seven participants answered in the negative for “I felt like the body was my own body” in both the PC and NC. In addition, whilst a control question of “I felt I had two bodies” was in the expected negative direction in terms of the median, the IQR included neutral or positive responses suggesting for some participants there was an experience of having two separate bodies and therefore successful embodiment may not have taken place (two participants for the positive condition and five participants for negative condition). There were no significant differences between conditions in embodiment (*Z Scores(19)<−1.8, ps* > .07) except for two questions (“I felt myself responding to the mother regarding my emotions” and “I felt like I was in the child’s body”) where there was significantly higher agreement with the statements following the PC compared to the NC (*Z(19)* = −2.1, *p* = .04) and *Z(19)* = 2.1, *p* = .04 respectively).

### Adult Adolescent Parenting Inventory

#### Scale reliabilities.

Scale reliabilities (Cronbach’s Alpha) for the AAPI overall (varied across time points from .71 to .84) and Inappropriate Parental Expectations (.58 to .84) were acceptable. Scale reliabilities for Parental Lack of Empathic Awareness (.41 to .68) and Strong Belief in the Value of Corporal Punishment (.41 to .71) were low and would fall under the threshold of 0.6 for comparing means suggested by Salvia and Ysseldyke [48] and therefore results relating these scales should be interpreted cautiously as they may not be measuring a unified construct. The scales for Parent-child Role Reversal (.06 to .67) and Oppressing Children’s Power and Independence (−1.0 to .31) were particularly poor and therefore results relating to these scales are not reported. The internal consistencies for each AAPI construct and overall score over the different time points are presented in S7 File.

#### Time A to Time D change.

Raw scores (Standard Ten (STEN) scores are presented in S8 File, the results of change in STEN scores followed the same pattern as the Raw scores) for the separate constructs and overall for Time A and Time D for each mother order condition are presented in Table 3. At Time A (baseline) mean raw scores generally scored at the upper end of the potential range (suggesting a low level of risk for the sample as a whole) and this remained the case at Time D.

**Table 3:** AAPI Mean Raw Scores (N= 18).

		Positive then Negative				Negative then Positive			
Construct		Time 1	Time 2			Time 1	Time 2		
	Range	*M (SD)*	*M (SD)*	*t (df = 7)^a^*	*p*	*M (SD)*	*M (SD)*	*t (df= 9)^a^*	*p*
Inappropriate parental expectations	7 - 35	28.5 (3.9)	29.5 (4.0)	-0.88	.40	27.4 (4.7)	26.2 (5.0)	1.48	.17
Parental lack of empathic awareness of children’s needs	10 - 50	47.4 (2.1)	47.4 (2.3)	0.00	1.0	44.9 (3.2)	44.8 (3.7)	0.15	.89
Strong belief in the use and value of corporal punishment	11 - 55	51.0 (4.3)	52.1 (2.4)	-0.97	.36	48.6 (4.3)	47.3 (4.3)	1.90	.09
Parent-child role reversal	7 - 35	28.0 (2.3)	29.3 (3.4)	-0.88	.44	29.6 (3.2)	28.2 (3.9)	1.95	.08
Oppressing children’s power and independence	5 - 25	19.6 (2.3)	20.6 (2.3)	-1.5	.19	22.2(1.8)	22.6 (1.8)	-1.31	.22
Overall Scale	40 - 200	174.5 (9.7)	178.9 (8.7)	-1.37	.21	172.7 (11.5)	169.1 (12.8)	1.89	.09

*Notes.* Mean raw scores (and standard deviations) for the AAPI at time one and time two split by the order in which participants experienced the two mother conditions. Higher scores reflect a lower level of child abuse risk.

^a^Non-parametric tests resulted in same results

Empathy (Construct B) scores were already very high at baseline. There was no change in Empathy from before to after exposure to both conditions (F(1, 16) = 0.013, *p* = .91, *ηp*^*2*^ = 0.001) and this did not interact with mother order (F(1, 16) = 0.013, *p* = .91, *ηp*^*2*^ = 0.001).

**Fig 2:**
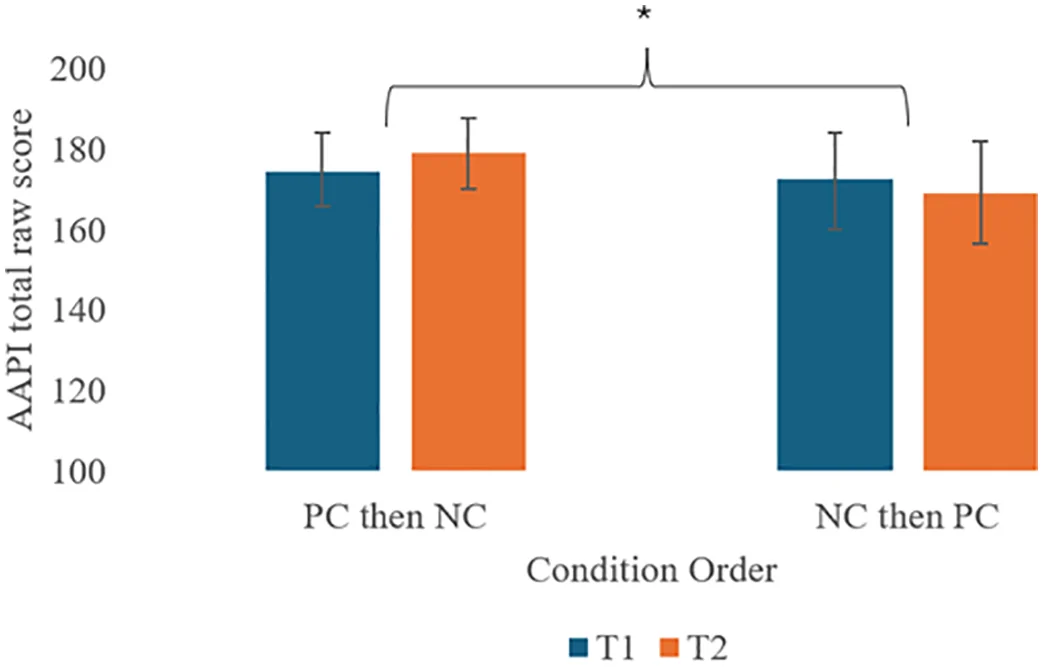
Total AAPI Raw Scores.

For Parental Unrealistic Expectations (Construct A), Value in the use of Corporal Punishment (Construct C) and Overall, participants scores did not change significantly (*F*s(1,16) ≤ 0.05, *ps* ≥ .83) from Time A to Time D. For Overall score, there was a significant interaction such that for PC then NC there was an increase in score (reflecting reduced risk) from Time A to Time D while for NC then PC there was a slight decrease (*F*(1,16) = 5.04, *p* = .04, *ηp*^*2*^ = 0.24; See Fig 2). There was no interaction between Time and Order for any other constructs (*F*s(1,16) ≤ 2.84, *p*s ≥ .11).

Fig 2 depicts the significant interaction between order of conditions and change in Total Raw Score for the AAPI from Time one (T1) to Time two (T2). Change from T1 to T2 was not significant for each condition separately. PC = Positive Mother Condition. NC = Negative Mother Condition

*p < .05

#### Relationship with attachment.

The scale reliability for ECR-R was high (Cronbach *a* = .88). Participants’ scores for attachment anxiety (*M = 2.4, SD = 0.8)* and avoidance (*M* = 2.3, *SD* = 1.0) were low, suggesting high attachment security in the sample as a whole. In terms of the relationship between attachment and change over time, there were no significant correlations between any of the AAPI constructs (including Empathy) or Overall score and attachment avoidance or anxiety (*r*s (18) between −.30 and .42, *ps* ≥ .08) The pattern of results for non-parametric correlations were consistent with this. The relationship between attachment anxiety and avoidance and scores for the AAPI at baseline are presented in S9 File.

### Parenting practices

#### Scale reliabilities.

The internal consistencies (Cronbach’s Alpha) for Parenting Scale subscales were generally acceptable for Laxness (.54 to .80), Overreactivity (.83 to .92) and Overall, (.70 to .81). Scale reliability for Verbosity was low (0.32 to 0.63; consistent with previous research [41,49]) and therefore results related to this scale should be interpreted with caution (Scale reliabilities are reported in full in S10 File).

#### Change over time.

There was no interaction between change and order of mother conditions for any of the parenting subscales at any time point (*F*s(1, 17) ≤ 0.36, *p*s ≥ .56) therefore differences between pre- and post-VR at the different time points are presented in Table 4 with orders merged.

**Table 4:** Mean Parenting Scores at Each Time Point Pre and Post VR.

Subscales	Time 1				Time 2				T1pre vs T2post	
	*M (SD)*		*Z*	*p*	*M (SD)*		*Z*	*p*	*Z*	*p*
	Pre	Post			Pre	Post				
Laxness	2.8 (0.8)	2.5 (0.5)	-1.6	.10	2.5 (0.6)	2.2(0.8)	-2.0	.04	-3.0	.003
Over-reactivity	2.3 (0.8)	1.8 (0.7)	-2.7	<.01	2.1 (0.8)	1.4 (0.7)	-3.7	<.001	-3.4	.001
Verbosity	3.9 (0.7)	3.8(0.8)	-0.9	.37	3.6 (0.8)	3.5 (0.9)	-0.9	.36	-2.3	.02
Total	3.0(0.5)	2.7 (0.4)	-2.3	.02	2.7 (0.5)	2.4 (0.6)	-2.6	.01	-3.5	<.001

*Notes.* Means and standard deviations of scores on the Parent Scale. Lower Scores represent more effective parenting practices. The distribution of scores were high in Skew and/or Kurtosis therefore results of non-parametric, Wilcoxon signed ranks are reported (although results of parametric tests followed the same pattern).

Participants’ scores reduced from Time A to B and Time C to Time D for Total score, Laxness (Time C vs Time D only) and Overreactivity (z-scores > −2.01) reflecting a move towards less dysfunctional parenting practices, but not for Verbosity (z-scores < −0.9). When comparing Time A to Time D, to account for effect of both conditions in combination, there was a significant reduction in score for Total score and all three subscales (*ts*(18) ≥ 2.64, *ps ≤* 0.02). There was no significant interaction between change in score and mother condition for any of the subscales or Total score (*F*s(17) ≤, *ps¸* ≥ .12).

#### Relationship with attachment.

For change in Parenting Scale scores over time, there was a significant negative correlation between attachment anxiety and change in Laxness at Time C vs Time D (τ_b_ = −0.36, *p = .*03) such that higher attachment anxiety resulted in less change towards more effective parenting practices, whilst there was also a significant negative correlation between attachment avoidance and change in Total score between Time A and Time D(τ_b_ = −0.33, *p <* .05) such that those higher in attachment avoidance reported less change towards more effective parenting practices. No other correlations were significant. Correlations between attachment scales and baseline Parenting Scale scores are presented in S9 File.

### Cognitive empathy (REMT)

The mean proportion of correct responses on the REMT are presented in Table 5. No significant difference between scores were found comparing any time points (*F*s(1,13) ≤ .21, *p*s ≥ .64) or the order participants were exposed to the PC and NC (*F*s(1,13) ≤ 0.08, *ps* ≥ .78). When looking at the effect of the PC and NC separately there was no significant change in empathy following either condition (*t*(17) = −.32, *p* = .75 and *t*(17) = 0.04, *p* = .97, respectively). There was no significant relationship between attachment dimensions and change in REMT score (τ_b_ ≤ −.26, *ps* ≥ .15).

**Table 5:** Proportion of Emotions Identified Correctly.

	Time 1				Time 2			
	*M(SD)*		*t* (df = 8)	*p*	*M(SD)*		*t* (df = 8)	*p*
Order	Pre	Post			Pre	Post		
A B	0.57 (0.07)	0.58 (0.82)	-0.19	.85	0.61 (1.10)	0.58 (0.09)	1.07	.31
B A	0.64 (0.09)	0.65 (0.09)	-0.65	.54	0.64 (0.13)	0.66 (0.12)	-0.72	.49

*Notes.* Order A B reflects competing version A of the Reading Mind in the Eyes Test before exposure to the VR (Pre) and version B after exposure to the VR (Post). Order B A reflects completing version B before exposure to the VR (Pre) and version A after exposure to the VR (Post)

### Qualitative data

Thematic Analysis of participants’ interviews following the PC and NC resulted in the development of nine themes with underlying subthemes. These themes are described below with examples of quotes representing each theme presented in Table 6.

**Table 6:** Themes and Subthemes Derived from Thematic Analysis.

Theme	*n^a^*	Subtheme	Quote	
(1) Valuable Learning Experience	6	Reflecting on and discussing VR experience in week in between	*“Over the first couple of days I talked about it with friends, I talked about it with my husband…”*	
	5	Immersion being preferable to theoretical learning	*“I think these are the sorts of things I’m always every day analysing and reading, but when you see it in reality, it’s a lot easier to learn from than reading…”*	
			Negative Mother	Positive Mother
(2) Positive and negative conditions evoking congruent emotional, cognitive and physical responses	18	Emotional	*“It was a bit scary actually”*	*“It made me feel nice about myself”*
	13	Cognitive	*“I felt like she was annoyed because I had not done the things she wanted me to do…”*	*“I felt like she was genuinely asking what I wanted to do…”*
	7	Physical	*“I could not give the mother eye-contact”*	*“I wanted to get closer to her”.*
(3) Comparing and Contrasting Conditions	13	*NA*	*“after comparing both positive and negative experiences, I’m surprised how effective it is…”*	
(4) Noticing the Size Difference	13	*NA*	*“I mean looking up at her, I don’t know, I did feel like a child”*	
(5) Integrating Mother and Child Perspective	14	*NA*	*“it also made me realise how easy it might be to intimidate a child if we are not careful with our body language”*	
(6) Reflecting on Past Experiences	11	*NA*	*“I think that feeds into your own experience of being parented as well…”*	
(7) Reflections on How Parents may Change their Behaviour Based on this Perspective	16	Physical/Behavioural	*“My sense was that my boys wanted me down on the floor with them, and now I actually see why it is so important”*	
	11	Changes to content of information given to children	*“I think its easy as an adult to forget, and as a parent to forget that your child probably doesn’t understand or is able to ask or answer back”*	
(8) Mindful Interactions with Children	12	*NA*	*“Just thinking about it more, rather than it just rolling off the tongue, just thinking more, so just thinking about it, and relaying it and thinking what’s the best way I can do something”*	
(9) VR Challenges and Future Changes	4	Challenges to relatability	*“it would have to be really far in the future because I don’t have those sort of expectations with my own child at the moment because he is so little…”*	
	7	Incongruence between presented condition and how it was experienced	*“But I also noticed the face change, at first I didn’t like it, then she looked nice”*	
	10	Challenges to immersion and plausibility	*“I think if I had longer in the VR equipment it might have felt more real”*	
	4	Questionnaires rather than VR influencing reflections	*“I know it was in the questionnaire anyway, but I think I was a little bit more cautious about the way I presented myself”*	

*Notes.* NA = Not Applicable.

^a^*n is the number of participants’ interviews the theme was present in* (i.e., prevalence).

#### Valuable learning experience.

Participants discussed how they found the VR a valuable learning experience.

#### Reflecting on and discussing the experience with others.

Having taken part, five participants reported that they discussed the experience and related this to their own parenting practices with partners and friends.

#### Immersion being preferable to theoretical learning.

Six participants talked about how the VR experience further supplemented and brought to life previous parenting concepts they had learnt about on a theoretical level. They described valuing the chance to “practice” and “feel” the concepts they had previously read about.

#### Positive and negative conditions evoking congruent emotional, cognitive and physical responses.

Eighteen participants reported responses in emotional, cognitive and physical domains that were consistent with that expected for the positive and negative conditions.

##### Emotional response.

Emotions reported by participants in response to the negative mother included shame, loneliness, fear and anxiety, sadness and feeling intimidated. Emotions evoked by the positive mother included happiness, feeling soothed and relaxed as well as feeling loved.

##### Cognitive response.

Participants’ cognitive responses were also congruent with the interaction style of the mothers in both conditions. In response to the negative mother participants disclosed a sense of unfairness, whilst also thinking they were responsible for something or an inconvenience. Whilst in response to the positive mother, participants discussed how they trusted the mother and had a sense that they were being listened to by the mother who appeared interested in what they had to say.

##### Physical response.

Interacting with the mothers also led to a physical/behavioural response in seven participants. For the negative mother, participants reported how they felt physically threatened and as a result cut-off from the mother and became defensive. Whilst in response to the positive mother, participants reported wanting to seek close proximity to her.

#### Comparing and contrasting conditions.

Reports of 13 participants also demonstrated they were comparing and contrasting their experiences with the mothers in the two conditions, and it was this comparison that at times helped highlight what the participants felt was important in terms of engaging with children.

#### Noticing size difference.

An element of the VR experience that stood out for 13 participants and was common across both conditions was the size difference between their VR body and that of the virtual mothers. This was commented on by thirteen participants.

#### Integrating mother and child perspective.

The responses evoked by participants in interacting with the mother along with an awareness of the size difference suggested participants were experiencing the VR from a child’s perspective. Furthermore fourteen participants reflected on their experiences during the interview in such a way that suggested they were holding both the perspective of the child and their perspective as a mother in mind concurrently. Participants reflected on the difference between how a child and adult can respond in a given situation and showed an increased awareness of how their child might be experiencing an interaction with them as a mother. Two participants also reflected how a communication style does not need to appear overtly abusive to be experienced as threatening by a child, whilst four participants commented on the power difference between a mother and a child.

#### Reflecting on past experiences.

For eleven participants, exposure to the VR conditions led to reflections on past experiences which included their own experiences of being parented and how this may have influenced their own parenting behaviours. They also noticed similarities and differences between their own parenting styles and that of the virtual mother conditions. Whilst for four participants exposure to the positive mother reinforced to them what they were already doing well.

#### Reflecting how they might change their behaviour based on this perspective.

Most participants (n = 18) also discussed how this experience would change how they intend to interact with their child in the future. The theme can be broken down in terms of more physical elements of interacting with a child and an element relating to content of information delivered to a child.

##### Physical/behavioural changes.

Participants discussed an intention to change how they came across physically, including being more conscious of tone and volume of speech, physically lowering themselves to the child’s level when interacting with them, and having an overall intention of coming across more positively.

##### Changes to content of information given to children.

Participants also talked about an intention to hold their child’s level of understanding in mind and as a result altering how they provided information or instructions to their child. Participants commented on an intention to consider their child’s level of understanding, the amount of information they may be able to process, whilst also indicating they would be conscious of how much time they give their child to process information which had been given. They also expressed an intention to ensure they gave their child sufficient time to talk.

However, whist the majority said the VR would change how they came across, three participants, in response to the negative mother, stated that it would not change their behaviour going forward: *“I don’t think this week has changed anything I don’t think, like last week it had a bit of an effect on me but this week, I don’t think so”.*

#### Mindful (intentional cognitive and emotional awareness) interactions with children.

Twelve participants also talked of an intention to think more about their responses to children before acting in their day-to-day life in interacting with children. They discussed how they thought it would be important to take a moment to pause before responding in their interactions with children and in this time check in with their own emotions and ensure they are not having a negative impact on the interaction, whilst also ensuring they were considering the perspective of the child in the situation.

#### VR challenges.

Despite exposure to the VR generally being a valuable learning experience, evoking emotions and reflections that appeared to evidence an intention in participants to take the perspective of their children and motivating parenting changes, there were some challenges with the VR exposure. By gaining an understanding of these challenges it helps inform future changes to fine-tune the VR experience going forward.

##### Challenges to relatability.

Two participants discussed how the VR child appeared to be younger than their own children and therefore it made it harder to relate to how the virtual mother was interacting with the child. The need for similarity to one’s own children was further emphasised by one participant finding it very easy to relate due to similarities of the VR child and their own child: *“The child looked like my daughter, same hair colour as me, and this made me feel more connected”.*

##### Incongruence between presented condition and how the condition was experienced.

Some of the above themes demonstrate, for most participants, the conditions evoked the responses one would predict, however, some participants’ comments suggested that certain elements of the VR conditions were experienced as the opposite to what was anticipated. Participants at times talked about the positive condition as being anxiety provoking, especially in regards to her body language as she entered the room. Whilst the dialogue of the positive condition, at times, led participants to feel disappointed that they were asked if they would like to play together without that ever coming to fruition.

##### Challenges to immersion and plausibility.

Ten participants also spoke about difficulties either feeling fully immersed in the experience or difficulty believing the interaction with the mother. Some of the challenges to feeling immersed in the experience included a pre-occupation with the experimenter being in the room in real life and therefore feeling self-conscious (one participant). Others stated they needed more time in the environment (four participants), and relatedly, it feeling easier to feel immersed the second time (three participants) due to feeling less nervous about what to expect. Two participants also commented that it felt unnatural talking to the virtual mother and two others stated that they did not find the interaction with the virtual mother believable.

##### Questionnaires rather than VR experience influencing reflections.

Whilst many participants attributed the VR paradigm itself to being responsible for changes, four participants discussed how thinking about the statements on the questionnaires in the context of their wider experience facilitated reflections on their parenting style and potentially could have contributed to intention to change parenting behaviours.

## Discussion

In this study, mothers were embodied via VR in a child’s body and exposed to a warm and supportive (positive) and harsh and critical (negative) mother. Participants completed measures of self-reported embodiment, parent maltreatment risk (including empathic awareness), cognitive empathy and parenting and attachment style. Participants were also interviewed about their experience. Participant embodiment in the child avatar is a pre-requisite for subsequent analysis in VR paradigms. Results for the present study showed that embodiment may not have been successful for all participants and therefore the other results should be interpreted with this in mind. Scores tended to suggest high body ownership and agency for the majority of questions such as feeling like they were in a child’s body, movement being caused by own body movement and feeling like a child, however for a central question of “I felt the body was my own body” a median score of one and interquartile range of four suggested a number of participants disagreed with this statement. There were generally no significant differences in embodiment between the positive and negative conditions except for two questions where participants reported feeling more embodied and responding to the mother in regards to their emotions in the PC than the NC. This is consistent with previous research that has shown that negative affect (both in VR and real-life) can decrease body ownership) [50,51].

The present study did not replicate a previous finding [38] of an increase in empathy following exposure to an VR environment. There was no evidence how participants scored on dimensions of adult attachment avoidance and anxiety moderated how much the VR exposure resulted in changes in empathy or maltreatment risk across all constructs. The study did however find evidence that parents endorsed more effective parenting strategies following exposure to the virtual mothers and that higher scores in attachment avoidance was related to less change towards effective parenting strategies overall. Higher attachment anxiety was also related to less change away from a Lax parenting style. In general, results from the thematic analysis suggested the VR environment was effective in evoking cognitive, behavioural and emotional responses congruent with the mother condition which helped facilitate mothers’ experiencing a mother-child interaction from a child’s perspective. Participants reflected on this child’s perspective and their previous experiences of parenting or being parented. Most participants also expressed an intention to make changes to their parenting practices in the future. The interviews also drew attention to problems with immersion and relatability to the mother conditions and elements that were experienced as incongruent in some participants and therefore may require some adjustment in future studies.

For both the self-report measure of empathy (AAPI) and cognitive empathy (REMT), we found no evidence that exposure to the VR mothers led to an increase in parents’ level of empathy. However, the AAPI empathy construct had poor internal consistency for the current sample, whilst participants were also likely already scoring at ceiling for this measure (scoring average of 46 out of a maximum of 50) and therefore it is difficult to draw strong conclusions as to the effectiveness of the VR in increasing empathy. In addition, our sample size did not meet that determined by power analysis, which increases the risk of a type II errors. However, Hamilton-Giachritsis et al. [38] found a difference in change in empathy between before and after the VR mother using similar sample size which may undermine this explanation. Internal consistencies were also poor for the other constructs of the AAPI, therefore conclusions drawn are limited and in particular for Oppressing Power and Independence. Hamilton-Giachritsis et al. [38] found this construct significantly increased in risk following VR in their study. The results of this study could suggest that results of Hamilton-Giachritsis et al., [38] should be interpreted cautiously due to the poor internal consistencies of the AAPI (although we do not know the internal consistencies found in their study). Hamilton-Giachritsis et al., [38] also used the standard pre- and post-versions of the questionnaire at pre- and post-VR experience and therefore any changes in scores found may be a result of inconsistencies between the forms. They also used a Spanish version whereas the current study used an English language version. In addition, closer inspection of the items which make up the constructs of each form indicate that not all items contributing to a construct demonstrate good face validity (see S11 File for detailed discussion) which is why we used the same questionnaire at pre- and post- to better ensure the same attitudes were being measured at each time point. For Total score for the AAPI (which had good internal consistency we found a significant interaction between time (before and after both conditions) and the order the conditions were presented such that completing the positive condition followed by the negative resulted in a reduction in risk, whilst completing the negative and then the positive resulted in slight increase in risk (the independent effects were not significant). Interestingly, Hamilton-Giachritsis et al., [38] only found an improvement in cognitive empathy for the negative condition only. Given this, it is possible that the result in the present study represents a recency effect of having been exposed to the negative mother second. A further possibility is highlighted by some participants’ comments on the positive condition during the qualitative interview. Participants commented that the positive mother initially had an intimidating facial expression at the start of the paradigm which then quickly changed to positive and therefore this may have reduced the impact of the positive condition or resulted in an inconsistent emotional response in the participant. Participants also commented on the fact that when they were exposed to the negative condition first, they had an expectation that the mother would be negative during the positive condition, consistent with participant accounts in Hamilton- Giachritsis et al. [38]. Therefore for participants who experienced the negative mother first, the second, positive condition became contaminated which may have reduced the overall effectiveness of the paradigm. This could suggest that having a different mother avatar for each exposure could help reduce this contamination. Notably, there was a week between the two exposures to the mother conditions, which appears to have been too short an interval to eliminate carryover effects from the first condition. This also may indicate that the negative mother effects are long lasting.

Parenting styles were also measured by the Parenting Scale [41] in the present study. Using this measure, change from before to after both VR conditions resulted in a significant reduction in self-reported dysfunctional parenting practices. The largest change appeared to be in Over-reactivity which perhaps reflects exposure to the negative mother and subsequently not wanting to replicate such a style with their own children. The qualitative results also provided evidence of participants’ intentions to change their parenting style. Whilst reflecting on their own styles during the interview, participants also shared that they talked to close friends and relatives about their experience and how they might change their behaviour based on it. They discussed how they would try to consider the child’s developmental level in terms of their ability to understand certain information, how they came across emotionally to the child and wanting to get physically down to the child’s level. Participants indicated an intention to become more aware of their own emotional state before interacting with their child and how this may influence their interaction and therefore how it would be experienced by the child. These reflections share similarity to definitions of empathy. For example, Decety and Jackson (2004) [52] define empathy as “the subjective experience of similarity between the feelings expressed by the self and others without losing sight of whose feelings belong to whom” (pg. 71). They consider that empathy is made up of three functional components that dynamically interact to produce the experience of empathy: (1) Affective sharing between the self and other, (2) self-other awareness such that the emotions of self and other are not confused and (3) mental flexibility to adopt the subjective perspective of another. In the present study, the qualitative data provides some evidence that participants were able to reflect on how they felt themselves in the paradigm (1) whilst also transposing this to how their child would feel in their interactions with them as parents (3). Participants also indicated an intention to become more aware of their own emotional response before interacting with their children so as to not let this impact on the interaction (2). However, reporting such an intention does not mean that participants would necessarily be successful in such an approach. Objective measures of behavioural change were not used in the present study but future studies should include such measures.

### Relationships with Attachment Anxiety and Avoidance

We found no relationship between anxiety or avoidance attachment dimensions and change in empathy over time. In terms of other parenting constructs, there were mixed findings. Where there was a significant relationship it tended to follow a pattern of: the higher the attachment anxiety or avoidance, the less change away from dysfunctional parenting practices. Specifically, there was less change in Laxness at Time one following VR exposures for participants higher in attachment anxiety. Such a pattern may make theoretical sense given attachment anxiety is characterised by fear of rejection. It is possible that being firm with boundaries with children (which is opposed to Laxness) may arouse parents’ fear of being disliked and therefore find it hard to move toward this style. There was less change overall in the Parenting Scale for individuals higher in attachment avoidance. This is perhaps in line with the theoretical underpinning described in the introduction such that those with avoidant attachment styles may disengage the caregiving system when presented with threat [53] and therefore this may have resulted in the VR environment having less of an impact on such individuals. However, the lack of a consistent change and the small sample size, along with the relatively low score for avoidance and particularly anxiety [15] for the sample as a whole means that results should be considered preliminary. It is possible that better representation of individuals high in attachment anxiety/avoidance could result in different findings. In making some tentative links to the components thought to contribute to empathy above [52], it is possible that those with high attachment anxiety may have difficulty with self-other awareness, i.e., a child’s emotions evoking emotions in themselves and then their response is as if the emotions belonged to them, whereas those in attachment avoidance may find affective sharing and perspective taking difficult, as soon as a threat occurs they “switch off” their perspective-taking and their capacity to feel what others are feeling.

### Implications

Direct comparison to the previous study by Hamilton-Giachritsis et al., [38] is difficult due to a number of differences between that study and the present study, such as using a British sample (therefore new English dialogue and voice actor, English wording of the AAPI as opposed to Spanish), and repeating the same AAPI Form version across time points. Importantly there also appeared to be difficulties with VR embodiment and plausibility in the present study. The presented pilot study did not replicate the findings of increase in maternal empathy following the VR experience. Nevertheless, there was partial corroboration with the earlier study, in that both studies observed a greater reduction in parenting-related risk when participants were exposed to the negative condition second. Further, results from the interview suggest participants found the VR experience a good learning opportunity and their responses and reflections were in-line with increased perspective-taking and they reflected on processes thought to be important in the experience of empathy [52].

Hamilton-Giachritsis et al., [38] also found that risk, as measured by the Power and Independence factor, increased in their study. The internal consistency of this factor in the present study was very low and therefore could not be interpreted. Hamilton-Giachritsis et al. [38] used a different Form (A) before the VR and after (B) and items contributing to the constructs appear to differ in face validity (S11 File) between the forms which may explain the change in risk in that study.

The problems found with the AAPI scale reliabilities in our study are line with a study by Connors et al. [40] who found low internal consistencies in the constructs and were not able to replicate the original factor structure. The sample in that study was a low income, rural population whose children were enrolled onto the Head Start program [54] and therefore very different to the current sample, but perhaps suggests the scale has problems with reliability across different demographic groups (see Moon, [55] for a further example of this and Yoon [56] and Yoon [57] for further exploration of the validity of risk of parent maltreatment measures). It is also possible British participants in the present study differed to the Spanish participants in Hamilton-Giachritsis et al. [38] in response tendencies. Research has demonstrated cultural differences in response tendencies to Likert scales (although there appears to be no research on the difference between British and Spanish participants specifically). For example, Lee et al. [58] found US nationals tended to be more likely to use extreme ends of a Likert scale compared to Japanese and Chinese participants, whilst Hui and Triandis [59] found Hispanics were more likely than non-Hispanics to use extreme ends of a scale.

Whilst not replicating the previous finding of changes in empathy, the current study did find a reduction in self-reported dysfunctional parenting attitudes, particularly for Overreactivity. Higher scores on this measure have been associated with increased child abuse potential [60] and higher parent child-aggression [61]. Given that reducing dysfunctional parenting practices is the overall goal of the VR environment exposure, the current study has provided some preliminary support for using such a methodology for reducing parenting practices associated with risk of child maltreatment. However, it should be acknowledged that the scale has not been validated for participants responding with how they intend to parent in the future, which was how it was operationalised in this study. Additionally, studies have shown that there is not always good concurrent validity between self-reported parenting practices and observed parenting practices [62].

As reported above, there was some preliminary evidence for the potential impact of the paradigm to be moderated by attachment style (holding all the caveats in mind of generally low mean attachment anxiety/avoidance scores and a sample high in education and socioeconomic status), therefore future research may need to account for attachment style in operationalising the VR paradigm with reference to attachment theory. Before this, studies with a larger sample size and better representation across the secure/insecure attachment and socioeconomic spectrum are needed. In addition, with better representation of those higher in attachment insecurity, one could examine qualitative feedback in response to the VR environment and if this differed in a systematic way to those higher in attachment security.

### Limitations and future research

In addition to the limitations already discussed, during the post-experience interviews, while many participants responded in-line with feeling embodied and emotions congruent with the virtual mother’s actions, some participants had difficulties feeling fully immersed in the experience and raised some interesting points that warrant further consideration in refining the VR paradigm. Some participants reported feeling scared of the positive mother initially as a result of both the previous exposure to the negative mother (as was also reported in Hamilton-Giachritsis et al., 2018) and that the positive mother momentarily displayed an intimidating facial expression as the paradigm began. Some participants also discussed that whilst the tone of the virtual mother was warm in the positive condition, her actions were not necessarily consistent with what she said (e.g., asking if the participant wanted to play together but then not playing). Participants also discussed that they did not have enough time to fully acclimatise to the environment and needed longer, whilst others felt self-conscious responding verbally to the mother, knowing that the experimenter was listening. These factors will need further consideration in the design of future studies using such a paradigm.

There were also aspects of the paradigm that emerged from participants’ interviews that are important to retain, including the exposure to both the positive and negative mother to provide a contrast of styles to reflect on (but perhaps having different avatars for each mother condition to reduce contamination between conditions), the clear size difference between the mother and child avatar, and the voice actor’s tone of voice. Interestingly, more participants reported difficulties with embodiment during the negative condition. One could speculate that situations that are experienced as too stressful during VR immersion result in problems with embodiment due to participants employing psychological strategies to cope with such a situation (such as avoidance), but this requires most robust exploration.

The study was not designed to examine whether particular demographic factors were associated with reduced immersion in the VR paradigm (although please see S9 for post-hoc correlations between demographic characteristics and self-reported immersion). This represents an important direction for future research, as identifying such predictors would help clarify for whom VR interventions are most effective and guide adaptations to increase suitability for individuals with certain characteristics. Notably, our post-hoc analyses revealed no relationship between attachment security and responses to any of the immersion items. Beyond demographic factors, future work should also consider other potential moderators of immersion, including psychological variables. For example, feelings of self-consciousness whilst in the lab environment may meaningfully influence immersion, warranting further systematic investigation. Future research with a larger, more heterogeneous sample is required.

The current difficulties with AAPI reliability and validity (and previously reported difficulties, e.g., [40]), highlights the importance of adequate checking of the face validity of items contributing to a construct when selecting a measure to use. It is possible items not appearing to represent well the construct to which they belong impacted on the reliability of the measure. Further, it highlights the importance of avoiding allocation of items to a construct on statistical terms alone when developing a measure, which may have been the case with the AAPI. It is recommended that in future research an alternative measure of maltreatment risk is used alongside the *total* AAPI score (which has shown good reliability), and the same AAPI form should be used both before and after due to a lack of consistency between the forms.

Further, while the adult version of the Mind in the Eyes test has shown consistent reliability and validity across studies [42] the child version has been less widely used and appears to have only been used once before for adults identifying child faces [63], as in the current study. In addition, while it has shown good ability to discriminate between known groups who are low in cognitive empathy (i.e., Autism Spectrum Disorder vs Controls [42]) it is less clear on the measure’s sensitivity to pick up changes over time. Many measures of cognitive and emotional empathy have been developed [64] however most self-report measures may be better characterised as measure of trait empathy rather than state empathy and it is unclear how good these measures are at picking up changes over time. It may be that physiological measures of affective empathy are good candidates to measure empathy in response to specific situations (e.g., using EEG, fMRI, eye-tracking or skin conductance; [65,66]) and to pick up changes over time, although these methods themselves have a number of limitations [64] and, therefore, using a combination of measures at present may be most appropriate. Further, future studies in the area should also look to measure parenting behaviours in the months following the VR experience to see if reported reductions in dysfunctional parenting practices as found in the present study are enacted in life.

## Conclusions

The present pilot study, based on a small, predominantly homogeneous, highly educated, high-SES sample, did not replicate the previous finding [38] of increased empathy after embodiment as a child interacting with positive and negative virtual mother. Furthermore, no associations emerged between changes in empathy and adult attachment anxiety or avoidance. The lack of replication is of value in that it suggests changes in maltreatment risk documented in the original study could be attributable to issues with reliability and a lack of consistency between pre- and post-versions of a measure used to measure maltreatment risk. This requires further investigation. We did find preliminary evidence that the paradigm resulted in, an intention at least, to engage in less dysfunctional parenting practices in the future, with some evidence of a relationship between the extent of this change and adult attachment style.

As discussed above, empathy appears to be a difficult concept to conceptualise and genuine changes in empathy may be difficult to measure. Given that the overall goal of paradigms is to induce behaviour change that will result in less dysfunctional parenting practices. It may be that it would be more fruitful and also more valid to measure actual behaviour change after such paradigm rather than mediating factors such as empathy. One could hold in mind the Golden Rule Embodiment Principle which other research has begun to focus on [34]. Behaviour could be measured via self-report or observation sometime after exposure to VR conditions, or as in recent studies, participants’ behavioural response (parenting style) to situations of interest could be measured within the virtual reality environment itself [67,68].

## Supporting information

S1 File Supplementary details of VR equipment and paradigm. (DOCX)

S2 File Video depicting sample of calibration procedure and participant interaction with negative and positive mother whilst in VR paradigm. (MP4)

S3 File VR body ownership questionnaire. (DOCX)

S4 File Qualitative Semi-structured Interview Guide. (DOCX)

S5 File Testing of parametric assumptions. (DOCX)

S6 File Responses to post-experience embodiment questionnaire. (DOCX)

S7 File AAPI Internal Reliability Statistics. (DOCX)

S8 File Mean Standard Ten (STEN) Score for AAPI. (DOCX)

S9 File Additional Correlation Tables. (DOCX)

S10 File Parenting Scale Internal Reliability Statistics. (DOCX)

S11 File Exploration of AAPI Construct Validity. (DOCX)
