# Supplementary material for: Exploring the moderating role of maternal attachment style on the impact of a virtual reality environment on parental empathy and style: A pilot study
Source: PLoS One. 2026 Aug 24;21(8):e0355199. doi: 10.1371/journal.pone.0355199 (PMC13502691; doi:10.1371/journal.pone.0355199)
Supplement: Supplementary file 1 [file pone.0355199.s001.docx]

**Supplementary Information S1: Supplementary details of VR equipment and paradigm**

**Equipment.**

The study used a HTC VIVE™ headset, three VIVE body motion trackers (one for the torso and one on each foot) as well as two VIVE controllers for hand and arm movements. The headset, body trackers and controllers communicated with two VIVE base stations using infrared technology to determine their position in virtual space. The paradigm was programmed using Unity Software. Participants could move around a 3x5m area within the lab if they wished to.

**The Virtual Room.**

The Virtual room consisted of a large mirror, a sofa, a large window with views of a city townhouse opposite in the daytime, a lamp, some books and a mug on a coffee table, a children’s play mat and some toys (a rocking horse, an origami bird, a train set and a ball).

**The Virtual Mother(s)**

The virtual mother had the same overall appearance for both Negative and Positive conditions but they differed in their facial expressions, body language, tone of voice and words communicated to the participant. The dialogue for the Negative and Positive conditions can be found in Table 3.1 and was the same as that used in Hamilton-Giachritsis et al. (2018) but translated to English. In short, the Negative mother was critical, blaming and authoritarian towards the child whilst the Positive mother was caring, praising and took a more collaborative approach encouraging the participant to help rather than demanding the child tidy up (as in the Negative condition). The pre-recorded sound (the same person for both Positive and Negative conditions) was provided by an English female who was rated in a pilot study (*n* = 7) as highest for warmth (*M*=7.8 out of 10) for the Positive and harshness (*M* =7.1 out of 10) for the Negative mother out of a pool of six voice actors. In the Negative condition the arms of the mother moved relatively vigorously and her facial expression was one of anger whilst, the Positive mother’s arm movements were slower and more gentle and had a happy (soft smile with mouth closed) facial expression.

| **Table S1. Virtual Mother Dialogue for Positive and Negative Condition** | |
| --- | --- |
| Positive mother | Negative mother |
| *[Mother*]: Hi darling how are you? *[Mother*]: You are not bored, are you? *[Mother*]: Do you want to play together for a while? *[Mother*]: Come on, what toy do you like the most? *[Mother*]: That is a very nice toy, I like it too. Tell me what is it that you like about this toy? [*Mother*]: You are right. Um, I’ll go and start making dinner and then perhaps we can go for a little walk together - we are going to have fun! [Back up phrase: don’t you want to come out for a walk with me?] *[Mother*]: Well, then I’ll see you in a bit? [*Mother*]: In the meantime, would you mind tiding up the room here a bit to help me out, please? I am very tired and that would be such a big help for me. [*Mother*] What a sweet girl you are! I love you so much! You always help me out, I’m very proud of you and I love spending time together. So does your daddy, maybe when he comes back he can be with us and we can play a game together. | [*Mother*]: There you are, what are you doing?  *[Mother*]: Did you call me? I am sure I heard you saying something  *[Mother*]: don’t bother me; can’t you see I am busy?  *[Mother*]: Don’t you like any of these toys?  [*Mother*]: You are making too much noise! Stop making noise.  [*Mother*]: Look, I’m going to make dinner and then we are out of here. I’ve had enough being inside here.  *[Mother*]: Once I go, tidy up this room. You have left everything untidy. The room looks a disaster. For God’s sake, why can’t you do what I am asking you for once? I am tired of doing everything around here without help and on top of that it’s you who disorganizes everything, who doesn’t do anything. What are you looking at? Tidy up! Now  [*Mother*]: You are such an untidy child. Why do I have such a naughty daughter? Why don’t you do what I ask for once? Is it too much to ask to leave me in peace now and then? Leave me in peace! Really, you are such a stupid girl, just like your father, stupid. Wait until he gets home and he hears how horrible you’ve been today, you will see! |

**The Child Body**

Participants were embodied in that of a white four year old virtual female body. The child is approximately one third of the height of virtual mother.
