# Supplementary material for: Exploring the moderating role of maternal attachment style on the impact of a virtual reality environment on parental empathy and style: A pilot study
Source: PLoS One. 2026 Aug 24;21(8):e0355199. doi: 10.1371/journal.pone.0355199 (PMC13502691; doi:10.1371/journal.pone.0355199)
Supplement: Supplementary file 2 [file pone.0355199.s003.docx]

**S3 File. Body Ownership Questionnaire**

How much do you agree with the following statements about your experience? Each statement should be answered with:

-3 Disagree strongly

-2 Disagree

-1 Disagree somewhat

0 Neither agree nor disagree

1 Agree somewhat

2 Agree

3 Strongly agree

1. The I felt the virtual body I was looking down at was my own body.

Disagree	-3	-2	-1	0	1	2	3	Agree

2. I felt was looking at myself when looking in the mirror.

Disagree	-3	-2	-1	0	1	2	3	Agree

3. The felt the virtual body resembled my own (real) body in terms of shape, skin tone or other visual features.

Disagree	-3	-2	-1	0	1	2	3	Agree

4. I felt that I had two bodies.

Disagree	-3	-2	-1	0	1	2	3	Agree

5. I felt that the movements of the virtual body were caused by my own real body movements.

Disagree	-3	-2	-1	0	1	2	3	Agree

**Please turn over**

6. I felt the mother I saw was aware of my presence.

Disagree	-3	-2	-1	0	1	2	3	Agree

7. I felt myself responding to the mother
   a. About my thoughts

Disagree	-3	-2	-1	0	1	2	3	Agree

b. About my feelings and emotions

Disagree	-3	-2	-1	0	1	2	3	Agree

c. Physically

Disagree	-3	-2	-1	0	1	2	3	Agre

8. I felt like I was in a child’s body

Disagree	-3	-2	-1	0	1	2	3	Agree

9. I felt the mother acted as if I was a child

Disagree	-3	-2	-1	0	1	2	3	Agree

10. I felt much younger than I actually am

Disagree	-3	-2	-1	0	1	2	3	Agree

11. I felt like a child

Disagree	-3	-2	-1	0	1	2	3	Agree

12. Please write down any other comment or feeling you had regarding your experience.

13. Do you have any after-effects from the experiment (continue response onto next page if needed)?
