# Supplementary material for: Exploring the moderating role of maternal attachment style on the impact of a virtual reality environment on parental empathy and style: A pilot study
Source: PLoS One. 2026 Aug 24;21(8):e0355199. doi: 10.1371/journal.pone.0355199 (PMC13502691; doi:10.1371/journal.pone.0355199)
Supplement: Supplementary file 3 [file pone.0355199.s004.docx]

**S4 File. Qualitative Semi-structured Interview Guide**

1. How was that experience?
2. How did you feel when the mother was so angry/negative/ignored you?
3. How did you feel when the mother was warm?
4. Do you think that this experience will make you **think** more about how you respond to your own children?
5. Do you think that this experience will make you **behave** differently to your own children? Why?
6. (T2 only) Has the experience of the VR last week made you think about how you might respond to your child over the last week?
