# Supplementary material for: Exploring the moderating role of maternal attachment style on the impact of a virtual reality environment on parental empathy and style: A pilot study
Source: PLoS One. 2026 Aug 24;21(8):e0355199. doi: 10.1371/journal.pone.0355199 (PMC13502691; doi:10.1371/journal.pone.0355199)
Supplement: Supplementary file 4 [file pone.0355199.s005.docx]

**S5 File. Testing of parametric assumptions**

**Mind in the eyes**

Shapiro-Wilk statistics all W(18) > .90, *p*s > .06.

**Parenting scale**

The Shapiro-Wilk test indicated the assumption of normality was violated for The Verbosity factor at T1 pre (W(19) = .76, *p* < .001, the Reactivity factor at T1 post (W(19) = .85, *p* < .01, the Laxness factor at T2 pre (W(19) = .89, *p* < .05, and the Reactivity factor at T2 post (W(19) = .62, *p* < .001. Therefore Wilcoxon-rank sum non-parametric analyses were additionally run for analysis of difference in parenting scale before and after the VR exposure.

**Experiences in Close relationship Scale**

The Shapiro-Wilk test indicated the attachment anxiety subscale was normally distributed (W(19) = .96, *p* = .47), whilst the attachment avoidance subscale was not normally distributed (W(19) - .89, *p* =.03) therefore non-parametric correlations were additionally run for analyses including this variable.

**Adult Adolescent Parenting Inventory 2.1**

The Shapiro-Wilk test indicated The Parental Empathy (construct B) construct was not normally distributed at T1 or T2 (W(18) < .90, *ps* ≤ .05), and Parental Belief in Corporal Punishment (construct C) at T1 (W(18) = .87, *p* = .02 but not for any other constructs at either time point (Ws(18) > .92, *ps* > .11). Therefore non-parametric equivalents were also run for analyses involving these variables.
