# Supplementary material for: Exploring the moderating role of maternal attachment style on the impact of a virtual reality environment on parental empathy and style: A pilot study
Source: PLoS One. 2026 Aug 24;21(8):e0355199. doi: 10.1371/journal.pone.0355199 (PMC13502691; doi:10.1371/journal.pone.0355199)
Supplement: Supplementary file 5 [file pone.0355199.s006.docx]

| **Table S6. Responses to post-experience embodiment questionnaire** | | | |
| --- | --- | --- | --- |
|  | Positive | Negative |  |
| Statement | *Mdn (IQR)* | *Mdn (IQR)* | *p* |
| I felt the body was my own body | 1 (-1 to 3) | 0.6 (2.1) | .70 |
| I felt I was looking at myself in the mirror | 2 (-1 to 3) | 0.5 (2.1) | .07 |
| The movement was caused by my own body movement | 3 (2 to 3) | 3 (2 to 3) | .79 |
| I felt the mother was aware of my presence | 3 (2 to 3) | 3 (2 to 3) | .53 |
| I felt myself responding to the mothers regarding my |  |  |  |
| Thoughts | 2 (1 to 3) | 2 (0 to 3) | .23 |
| Emotion | 2 (1 to 3) | 1 (-1 to 2) | .04 |
| Physical response | 2 (0 to 3) | 1 (-1 to 2) | .12 |
| I felt like I was in a child’s body | 3 (1 to 3) | 2 (1 to 2) | .03 |
| The mother acted as if I was a child | 3 (3 to 3) | 3 (2 to 3) | .27 |
| I felt much younger than I actually am | 3 (1 to 3) | 3 (2 to 3) | .45 |
| I felt like a child | 3 (1 to 3) | 2 (2 to 3) | .58 |
| *Control questions^a^* |  |  |  |
| I felt the body resembled my own body in terms of features | 1(-3 to 2) | -1 (-2 to 3) | 1.0 |
| I felt I had two bodies | -2 (-3 to -1) | -1.2 (1.7) | .10 |
| *Notes.Mdn* = Median. *IQR* = Interquartile Range*.* Higher scores represent more agreement (more successful embodiment). *P values represent those conducted exploring differences between conditions using paired sample t-test.*  ^a^Control questions, where lower or negative scores show more successful embodiment. | | | |

**S6 File. Responses to post-experience embodiment questionnaire**
