# Supplementary material for: Exploring the moderating role of maternal attachment style on the impact of a virtual reality environment on parental empathy and style: A pilot study
Source: PLoS One. 2026 Aug 24;21(8):e0355199. doi: 10.1371/journal.pone.0355199 (PMC13502691; doi:10.1371/journal.pone.0355199)
Supplement: Supplementary file 6 [file pone.0355199.s007.docx]

**S7 File. AAPI Internal Reliability Statistics**

| **Table S7. *Cronbach Alpha’s for AAPI Subscales and Overall Scale*** | | | | |
| --- | --- | --- | --- | --- |
|  | Form A | | Form B | |
| Construct | Time 1 | Time 2 | Time 1 | Time 2 |
| A. Inappropriate parental expectations | .77 | .84 | .58 | .82 |
| B. Parental lack of empathic awareness of children’s needs | .48 | .68 | .41^a^ | .50^a^ |
| C. Strong belief in the use and value of corporal punishment | .71 | .41 | .47^a^ | .57^a^ |
| D. Parent-child role reversal | .59 | .67 | .06^a^ | .41^a^ |
| E. Oppressing children’s power and independence | .13 | -1.03^b^ | -.55 ^b^ | .31 |
| Overall Scale | .84 | .84 | .71^a^ | .79^a^ |
| *Notes.* AAPI = Adult Adolescent Parenting Inventory  ^a^Items automatically removed from scale due to zero variance  ^b^Coding was double checked to ensure items for coded in the correct direction | | | | |
