# Supplementary material for: Exploring the moderating role of maternal attachment style on the impact of a virtual reality environment on parental empathy and style: A pilot study
Source: PLoS One. 2026 Aug 24;21(8):e0355199. doi: 10.1371/journal.pone.0355199 (PMC13502691; doi:10.1371/journal.pone.0355199)
Supplement: Supplementary file 7 [file pone.0355199.s008.docx]

**S8 File. Mean Standard Ten (STEN) Score for AAPI**

| **Table S8 *STEN Scores for AAPI Before and After Exposure to Both VR Conditions*** | | | |
| --- | --- | --- | --- |
| Construct | Time 1 | Time 2 | *p* |
|  | M (SD) | M (SD) |  |
| A. Inappropriate parental expectations | 7.4(1.6) | 7.3 (1.9) | .53 |
| B. Parental lack of empathic awareness of children’s needs | 6.9 (1.7) | 7.1(1.7) | .48 |
| C. Strong belief in the use and value of corporal punishment | 7.0(1.5) | 7.1 (1.5) | .86 |
| D. Parent-child role reversal | 6.5(1.3) | 6.4 (1.7) | .88 |
| E. Oppressing children’s power and independence | 6.5(1.8) | 7.1(1.8) | .06 |
| *Notes.* AAPI = Adult Adolescent Parenting Inventory 2.1. Higher scores associated with lower risk. STEN = Standard Ten | | | |
