# Supplementary material for: Exploring the moderating role of maternal attachment style on the impact of a virtual reality environment on parental empathy and style: A pilot study
Source: PLoS One. 2026 Aug 24;21(8):e0355199. doi: 10.1371/journal.pone.0355199 (PMC13502691; doi:10.1371/journal.pone.0355199)
Supplement: Supplementary file 8 [file pone.0355199.s009.docx]

**S9 File. Additional Correlation tables**

| **Table S9a. C*orrelations between attachment subscales and Dependent Variables at baseline (T1)*** | | | | | | | | | | | | |
| --- | --- | --- | --- | --- | --- | --- | --- | --- | --- | --- | --- | --- |
|  | 1 | 2 | 3 | 4 | 5 | 6 | 7 | 8 | 9 | 10 | 11 | 12 |
| 1. Attachment Anxiety | - |  |  |  |  |  |  |  |  |  |  |  |
| 2. Attachment Avoidance | .53** | - |  |  |  |  |  |  |  |  |  |  |
| 3. AAPI Unrealistic expectations | -.31† | -.23 | - |  |  |  |  |  |  |  |  |  |
| 4. AAPI Empathy | -.30† | -.18 | .31† | - |  |  |  |  |  |  |  |  |
| 5. AAPI corporal punishment | -.22 | -.34* | .37* | .40* | - |  |  |  |  |  |  |  |
| 6. AAPI Role reversal | -.05 | -.12 | .25 | .40* | .21 | - |  |  |  |  |  |  |
| 7. AAPI power and independence | -.15 | .01 | -.05 | -.16 | -.15 | .15 | - |  |  |  |  |  |
| 8. AAPI Total | -.30† | -.28 | .57** | .53** | .61** | .53** | .06 | - |  |  |  |  |
| 9. PS Laxness | -.11 | -.01 | -.10 | -.12 | -.13 | -.32† | -.04 | -.19 | - |  |  |  |
| 10. PS Over reactivity | .04 | -.20 | -.19 | -.05 | .14 | -.11 | .01 | -.12 | .05 | - |  |  |
| 11. PS Verbosity | .45* | .34* | -.36* | -.14 | -.29 | -.25 | -.21 | -.50* | .04 | .30 | - |  |
| 12. PS total | .05 | <.01 | -.13 | -.11 | -.26 | -.25 | -.13 | -.26 | .60*** | .30* | .30† | - |
| 13. Mind in the eyes | .18 | .02 | .04 | .04 | .01 | .03 | -.37* | -.07 | .04 | .26 | .04 | .21 |
| *Notes.*  AAPI = Adult Adolescent Parenting Inventory 2.1. PS = Parent Scale. All correlations Kendall’s Tau  †p<.10 *p<.05 ** p< .01 ***p<.001. PS, Parent Scale All correlations Kendall’s Tau | | | | | | | | | | | | |

**Table S9** . Pearson correlations among demographic variables and embodiment questions in response to Warm VR condition

|  | Demographics | | | | | | Embodiment Questions | | | | | | | | | | | |
| --- | --- | --- | --- | --- | --- | --- | --- | --- | --- | --- | --- | --- | --- | --- | --- | --- | --- | --- |
| Variable | 1 | 2 | 3 | 4 | 5 | 6 | 7 | 8 | 9 | 10 | 11 | 12 | 13 | 14 | 15 | 16 | 17 | 18 |
| 1. Age |  |  |  |  |  |  |  |  |  |  |  |  |  |  |  |  |  |  |
| 2. Highest grade completed | .17 |  |  |  |  |  |  |  |  |  |  |  |  |  |  |  |  |  |
| 3. Household income | .62** | .40 |  |  |  |  |  |  |  |  |  |  |  |  |  |  |  |  |
| 4. Current employment status | .13 | -.42 | -.08 |  |  |  |  |  |  |  |  |  |  |  |  |  |  |  |
| 5. Participant or their partner in military | -.03 | -.18 | -.16 | .15 |  |  |  |  |  |  |  |  |  |  |  |  |  |  |
| 6. Victim of abuse from someone in family | .66** | .56* | .70** | -.11 | -.15 |  |  |  |  |  |  |  |  |  |  |  |  |  |
| 7. Body was own body | .06 | -.06 | -.16 | -.03 | .04 | -.20 |  |  |  |  |  |  |  |  |  |  |  |  |
| 8. Felt looking at self in mirror | -.11 | -.00 | -.29 | .00 | .06 | -.30 | .95** |  |  |  |  |  |  |  |  |  |  |  |
| 9. Resembled my own features | -.03 | .15 | .37 | .14 | -.16 | .25 | -.57* | -.65** |  |  |  |  |  |  |  |  |  |  |
| 10. Felt like I had two bodies | -.08 | .39 | .12 | -.76** | .05 | .12 | -.07 | -.11 | -.14 |  |  |  |  |  |  |  |  |  |
| 11. Movement caused by own body movement | .08 | -.06 | -.29 | -.25 | -.18 | -.22 | -.07 | -.11 | -.30 | .31 |  |  |  |  |  |  |  |  |
| 12. Felt other woman was aware of my presence | .09 | -.10 | -.03 | -.20 | .24 | -.12 | .24 | .18 | -.37 | .43 | .04 |  |  |  |  |  |  |  |
| 13. Felt self responded about my thought | .21 | .04 | .22 | -.01 | -.01 | .19 | .13 | -.06 | .17 | .04 | .20 | .30 |  |  |  |  |  |  |
| 14. Felt responding about my emotion | .20 | -.08 | .09 | -.17 | .15 | .09 | .17 | -.05 | .06 | .13 | .29 | .40 | .89** |  |  |  |  |  |
| 15. Felt responding physically | -.03 | -.03 | -.18 | -.30 | .24 | -.24 | .01 | -.05 | -.23 | .35 | .45 | .49* | .56* | .65** |  |  |  |  |
| 16. Felt like I was in child’s body | .03 | -.30 | -.07 | -.28 | .43 | -.22 | .13 | .04 | -.23 | .37 | .13 | .66** | .15 | .37 | .54* |  |  |  |
| 17. Felt the woman acted as if I was a child | .30 | -.11 | -.25 | -.32 | .15 | .06 | .08 | -.07 | -.27 | .36 | .70** | .34 | .40 | .62** | .57* | .49* |  |  |
| 18. Felt much younger than I actually am | .32 | -.15 | .13 | -.06 | .34 | .18 | .19 | .01 | -.16 | .17 | .14 | .63** | .58** | .72** | .32 | .46* | .55* |  |
| 19. Felt like a child | .27 | -.15 | .11 | -.10 | .12 | .20 | .20 | .05 | -.13 | .10 | .06 | .60** | .51* | .60** | .18 | .45 | .48* | .91** |

Note. Entries are Pearson correlation coefficients. Values above the diagonal are omitted. N = 19. * p < .05. ** p < .01 (two-tailed).

**Table S9c**. Pearson correlations among demographic variables and embodiment questions to Harsh VR condition.

|  | Demographics | | | | | | Embodiment Questions | | | | | | | | | | | |
| --- | --- | --- | --- | --- | --- | --- | --- | --- | --- | --- | --- | --- | --- | --- | --- | --- | --- | --- |
| Variable | 1 | 2 | 3 | 4 | 5 | 6 | 7 | 8 | 9 | 10 | 11 | 12 | 13 | 14 | 15 | 16 | 17 | 18 |
| 1. Age |  |  |  |  |  |  |  |  |  |  |  |  |  |  |  |  |  |  |
| 2. Highest grade completed | .17 |  |  |  |  |  |  |  |  |  |  |  |  |  |  |  |  |  |
| 3. Household income | .62** | .40 |  |  |  |  |  |  |  |  |  |  |  |  |  |  |  |  |
| 4. Current employment status | .13 | -.42 | -.08 |  |  |  |  |  |  |  |  |  |  |  |  |  |  |  |
| 5. Participant or their partner in military | -.03 | -.18 | -.16 | .15 |  |  |  |  |  |  |  |  |  |  |  |  |  |  |
| 6. Victim of abuse from someone in family | .66** | .56* | .70** | -.11 | -.15 |  |  |  |  |  |  |  |  |  |  |  |  |  |
| 7. body was own body | -.12 | -.05 | -.34 | -.29 | .02 | -.35 |  |  |  |  |  |  |  |  |  |  |  |  |
| 8. Felt looking at self in mirror | .03 | .03 | -.17 | -.14 | -.00 | -.11 | .90** |  |  |  |  |  |  |  |  |  |  |  |
| 9. body resembled my own features | -.02 | .11 | .33 | .08 | -.09 | .25 | -.87** | -.80** |  |  |  |  |  |  |  |  |  |  |
| 10. felt like I had two bodies | -.09 | .10 | .21 | -.46* | -.28 | .04 | -.03 | -.17 | .06 |  |  |  |  |  |  |  |  |  |
| 11. Movement caused by own body movement | .31 | .12 | -.16 | -.05 | -.27 | -.14 | .30 | .22 | -.34 | -.14 |  |  |  |  |  |  |  |  |
| 12. Felt other woman was aware of my presence | .19 | -.11 | -.17 | -.20 | .58** | -.11 | .39 | .33 | -.43 | .02 | .13 |  |  |  |  |  |  |  |
| 13. felt self responded about my thoughts | .15 | -.27 | -.23 | .03 | .26 | -.09 | .30 | .34 | -.32 | -.06 | .22 | .65** |  |  |  |  |  |  |
| 14. felt responding about my emotions | -.01 | -.35 | -.14 | .05 | .09 | -.11 | .19 | .26 | -.17 | .02 | -.01 | .43 | .78** |  |  |  |  |  |
| 15. Felt responding physically | .09 | .21 | .06 | -.09 | .28 | .05 | .25 | .47* | -.24 | -.21 | .05 | .49* | .41 | .51* |  |  |  |  |
| 16. Felt like I was in childs body | .09 | -.11 | -.04 | -.29 | .32 | -.11 | .46* | .33 | -.41 | .20 | .03 | .82** | .58** | .55* | .26 |  |  |  |
| 17. Felt the woman acted as if I was a child | .24 | -.26 | -.05 | -.34 | .27 | -.24 | .34 | .17 | -.37 | .18 | .41 | .69** | .51* | .23 | .20 | .53* |  |  |
| 18. Felt much younger than I actually am | .58** | -.12 | .30 | .00 | .33 | .28 | .11 | .08 | -.11 | -.10 | .09 | .59** | .24 | .21 | .04 | .65** | .36 |  |
| 19. Felt like a child | .50* | -.20 | .32 | -.04 | .20 | .19 | .04 | -.02 | -.02 | -.07 | .08 | .43 | .10 | .13 | -.12 | .60** | .33 | .93** |

Note. Entries are Pearson correlation coefficients. Values above the diagonal are omitted. N = 19. * p < .05. ** p < .01 (two-tailed).
