# Supplementary material for: Exploring the moderating role of maternal attachment style on the impact of a virtual reality environment on parental empathy and style: A pilot study
Source: PLoS One. 2026 Aug 24;21(8):e0355199. doi: 10.1371/journal.pone.0355199 (PMC13502691; doi:10.1371/journal.pone.0355199)
Supplement: Supplementary file 9 [file pone.0355199.s010.docx]

**S10 File. Parenting Scale Internal Reliability Statistics**

| **Table S10. *Cronbach's alphas for Parenting Scale Subscales (N=19)*** | | | | |
| --- | --- | --- | --- | --- |
| Subscale | Time 1 | | Time 2 | |
|  | Pre | Post | Pre | Post |
| Laxness | 0.78 | 0.54 | 0.64 | 0.80 |
| Overreactivity | 0.83 | 0.86 | 0.85 | 0.92 |
| Verbosity | 0.32 | 0.63 | 0.46 | 0.55 |
| Total | 0.78 | 0.76 | 0.7 | 0.81 |
|  | | | | |
