# Supplementary material for: Exploring the moderating role of maternal attachment style on the impact of a virtual reality environment on parental empathy and style: A pilot study
Source: PLoS One. 2026 Aug 24;21(8):e0355199. doi: 10.1371/journal.pone.0355199 (PMC13502691; doi:10.1371/journal.pone.0355199)
Supplement: Supplementary file 10 [file pone.0355199.s011.docx]

**S11 File. Exploration of AAPI construct validity**

The development guide of the AAPI defines the power and independence factor as:

*“The age old phrase “the terrible two’s” most adequately describes this construct. Parents fear that if children are permitted to use their power and independence to explore their environment, or ask questions, or challenge parental authority, they will become “acting-out” and disrespectful. Hence, obedience and complete compliance to parental authority is demanded. When children’s power and independence are oppressed, they are not allowed to challenge, to voice opinions, or to have choices, but rather are told to “do what they are told to do” without question”*

The items that most strongly related to this factor in the validation of this study can be found in the table below (recreated from AAPI development guide):

| ***Table S11 showing items contributing to factor E Power and Independence and their loadings on other factors. Table recreated from AAPI development Guide (Bavolek & Keene, 2010)*** | | | | | | |
| --- | --- | --- | --- | --- | --- | --- |
|  |  | Loadings on factors | | | | |
| Form | Item | Inappropriate expectations A | Empathy B | Corporal Punishment C | Role Reversal D | **Power and Independence E** |
| A | Parents who encourage their children to talk only end up listening to complaints | 0.24 | 0.31 | -0.09 | 0.16 | **0.34** |
| A | Children need to be allowed the freedom to explore their world in safety | -0.13 | -0.14 | 0.00 | 0.01 | **-0.45** |
| A | Parents who nurture themselves make better parents | -0.11 | 0.01 | -0.14 | -0.19 | **-0.42** |
| A | Children should be potty trained when they are ready and not before | -0.04 | 0.02 | -0.17 | -0.21 | **0.27** |
| A | Children who receive praise will think too much of themselves | 0.17 | 0.19 | 0.08 | 0.17 | **0.36** |
| B | Consequences are necessary for family rules to have meaning | 0.11 | -0.12 | 0.06 | -0.11 | **-0.44** |
| B | Children who learn to recognise feelings in others are more successful in life | 0.05 | -0.09 | -0.16 | 0.08 | **-0.45** |
| B | Parents expectations of their children should be high but appropriate | 0.10 | -0.20 | -0.03 | 0.09 | **-0.45** |
| B | Rewarding children’s behaviour is a good form of discipline | 0.12 | -0.19 | -0.13 | -0.07 | **-0.37** |
| B | Parents who are sensitive to their children’s feelings and moods often spoil them | 0.26 | 0.20 | 0.15 | 0.11 | **0.28** |

As can be seen from the table above, whilst some items do tend to have strong face validity with the definition provided above e.g. “Parents who encourage their children to talk to them only end up listening to complaints” and “Children need to be allowed freedom to explore their world in safety”, other items have a less clear connection with the factor definition e.g. “children should be potty trained when they are ready and not before”, “parents who nurture themselves make better parents”.

**Lack of Consistency Between Items Contributing to Constructs Across Form A and Form B**

As well as potential difficulties with face validity in terms of the items’ relationship with the description of the construct, there is also a lack of consistency in terms of similar items contributing to different constructs across Form A and Form B. For example, for Form A an item worded “Children should be potty trained when they are ready and not before” loads onto construct E (Oppressing power and Independence ) however a similar item from Form B “children should be potty trained as soon as they are two years old” loads onto construct D (Parent-child Role Reversal, which in addition seems to lack a clear correspondence with this construct). Similarly an item on Form A worded “Children need to be allowed freedom to explore their world in safety” loads onto construct E, whilst a similarly but oppositely worded item from Form B “The problem with kids today is that parents give them too much freedom”, loads onto construct A (Parental inappropriate expectations of children).
