# Supplementary material for: Exploring the moderating role of maternal attachment style on the impact of a virtual reality environment on parental empathy and style: A pilot study
Source: PLoS One. 2026 Aug 24;21(8):e0355199. doi: 10.1371/journal.pone.0355199 (PMC13502691; doi:10.1371/journal.pone.0355199)
Supplement: Supplementary file 11 [file pone.0355199.s013.docx]

Dear Editor,

Thank you for providing the opportunity to submit a revised version of the attached manuscript . I am grateful for the reviewers’ helpful comments and I believe addressing these comments has significantly strengthened the paper.

Please see below for information on the specific revisions that have been made since the previous submission.

Yours sincerely,

Jac Airdrie

**Review comments**

The article addresses an interesting topic. I have followed the authors’ work since the virtual environment was among the first adopted to study perspective-taking in VR with the paradigm of virtual embodiment. Applying the current VE to the study of attachment is ground-breaking. Although the clinical aspects of attachment are more complex and involve a broader range of emotional and psychological factors than empathy and perspective-taking alone, VR has shown potential as a tool to enhance these aspects and to address the issue of child maltreatment. However, the sample size is too small to allow for generalization of the results, although it could serve as a valuable pilot study. I recommend that the authors address the reviewers’ requirements before the article can be considered for publication.

We thank the editor for the above comment. In order for the reader to better understand the limitations preventing the implications being taken too strongly and that the findings shouldn’t be generalised, the title has been changed to include the word “exploring” rather than definitively determining the “impact” in relation to maternal attachment style. We have changed “efficacy” to “impact” as efficacy might suggest the study was done under controlled conditions but whilst we counterbalanced groups there was no proper control of participants who did not receive any aspects of an intervention. In addition, we have included “a pilot study” to demonstrate that this was early tentative and explorative work. In addition, in regards to the title, we have highlighted that the findings also relate to parenting “style” as both reviewers commented on the fact that this was an interesting element of the paper.

“Pilot study” has also been added to title and the abstract.

Added pilot study to line 137.

The nature of the results being preliminary was already highlighted in a number of places in the manuscript (see lines 687, 736, 802) but further reference to the results being preliminary has been added throughout (lines 43, 55, 151, 731)

[Note: HTML markup is below. Please do not edit.]

Reviewers' comments:

Reviewer's Responses to Questions

**Comments to the Author**

1. Is the manuscript technically sound, and do the data support the conclusions?

The manuscript must describe a technically sound piece of scientific research with data that supports the conclusions. Experiments must have been conducted rigorously, with appropriate controls, replication, and sample sizes. The conclusions must be drawn appropriately based on the data presented.

Reviewer #1: Partly

Reviewer #2: Yes

2. Has the statistical analysis been performed appropriately and rigorously?

Reviewer #1: Yes

Reviewer #2: Yes

3. Have the authors made all data underlying the findings in their manuscript fully available?

The [PLOS Data policy](https://eur03.safelinks.protection.outlook.com/?url=http%3A%2F%2Ftrack.editorialmanager.com%2FCL0%2Fhttp%3A%252F%252Fwww.plosone.org%252Fstatic%252Fpolicies.action%2523sharing%2F1%2F010f0198d148a938-c77dab24-6c50-48c9-a04c-c1b05754caba-000000%2FKyk1nJhmpci2CQz7RwB6MgwTJ3cTSsgpsPjI0vIF5bQ%3D225&data=05%7C02%7Cairdriej%40cardiff.ac.uk%7C55641e78413f4db7fe0508de11f94fc9%7Cbdb74b3095684856bdbf06759778fcbc%7C1%7C0%7C638967959938215152%7CUnknown%7CTWFpbGZsb3d8eyJFbXB0eU1hcGkiOnRydWUsIlYiOiIwLjAuMDAwMCIsIlAiOiJXaW4zMiIsIkFOIjoiTWFpbCIsIldUIjoyfQ%3D%3D%7C0%7C%7C%7C&sdata=P1YD416ZV2UDxfi%2B4yWHzkJhYpkUwb0K35NFaEJSDBc%3D&reserved=0) requires authors to make all data underlying the findings described in their manuscript fully available without restriction, with rare exception (please refer to the Data Availability Statement in the manuscript PDF file). The data should be provided as part of the manuscript or its supporting information, or deposited to a public repository. For example, in addition to summary statistics, the data points behind means, medians and variance measures should be available. If there are restrictions on publicly sharing data—e.g. participant privacy or use of data from a third party—those must be specified.

Reviewer #1: Yes

Reviewer #2: No

We have included the statement that data can be made available on request – we do not have the appropriate consent to make it available without restriction.

4. Is the manuscript presented in an intelligible fashion and written in standard English?

PLOS ONE does not copyedit accepted manuscripts, so the language in submitted articles must be clear, correct, and unambiguous. Any typographical or grammatical errors should be corrected at revision, so please note any specific errors here.

Reviewer #1: Yes

Reviewer #2: No

- The manuscript has been proof-read throughout with consistent use of capitalisation, brackets and statistical terms have been italicised.
- Lower case has been used to describe positive and negative mothers throughout except when used in tables.

5. Review Comments to the Author

Please use the space provided to explain your answers to the questions above. You may also include additional comments for the author, including concerns about dual publication, research ethics, or publication ethics. (Please upload your review as an attachment if it exceeds 20,000 characters)

Reviewer #1: Dear Authors
congratulations on the work you have done. I read it thoroughly and personally I enjoyed a lot and honestly, it enlighted many lamps in my mind!
However, in order to enhance the quality of your manuscript I add some comments you may find useful:

1- The abstract provides a clear overview of the study's objectives, methods, and key findings. It mentions the sample size, design, and primary outcomes, such as the lack of change in empathy scores and the improvement in parenting attitudes. However, it could briefly highlight the qualitative findings, which add depth to the quantitative results. The abstract is concise but might benefit from a sentence summarizing the implications for future research or practice.

We thank the reviewer for the feedback. The abstract has a brief summary of the qualitative findings, and while we agree it would be ideal to include more, it is challenging to expand on them further while keeping to the required word count. Nevertheless, to make it easier for the reader to identify these findings, the term “qualitative” has been added to the abstract and the wording has been revised to clarify that, during the qualitative interviews, participants self-reported changes to their parenting behaviours. We have also highlighted that we will discuss findings that suggest potential modifications to the paradigm that could improve immersion.

We have further qualified the evidence it provides from “provides evidence” to “some preliminary support…” (line 55)

A further statement has been added that highlights the implications of the research, specifically the need for further replication with a more representative sample and more robustly validated measures of cognitive and emotional empathy (line 49).

2. The study addresses an important topic—using VR to enhance parental empathy and reduce child maltreatment risk. The innovation lies in exploring how attachment styles moderate VR's effects. However, the study does not replicate previous findings on empathy changes, which limits its novelty. The focus on attachment styles adds value, but the small sample and homogeneity of participants reduce the generalizability of the results.

We appreciate the observation regarding the lack of replication of previous findings on empathy changes and the limitations posed by our relatively small and homogeneous sample. We agree that broader replication is essential, and we have now strengthened this point explicitly in the abstract, emphasizing the need for future studies with larger and more diverse participant groups to enhance generalizability.

At the same time, we believe the study maintains its contribution to the field. In particular, the preliminary finding that changes in self-reported parenting behaviour appear to be moderated by attachment style provides a novel angle that adds meaningful value and may motivate further research in this area. We have revised the manuscript to highlight this contribution more clearly.

We think it is also important to highlight that the sample size in the current study (n=19) is similar to that used in the previous Hamilton-Giachritsis et al., (2018) study (n = 21) which did find a difference in empathy. Possible alternative reasons for the lack of replication including key methodological differences (such as differences in culture/nationality of the sample) and a ceiling effect of empathy being found at baseline are discussed thoroughly in the manuscript and in response to comment 5 below.

3. The within-groups, counterbalanced design is appropriate for testing VR's effects. The use of both quantitative and qualitative methods strengthens the study. However, the small sample size (N=19) and lack of diversity (high SES, highly educated) are major limitations. The measures, such as the AAPI, had poor reliability for some constructs, which affects the validity of the findings. The study could have included behavioral measures to complement self-reports.

We thank the reviewer for acknowledging the strengths of our within-groups counterbalanced design and the mixed-methods approach. We also agree that the small sample size and limited diversity of participants are important limitations of the current study.

Regarding the reliability of the measures, we have already addressed this issue in detail in the manuscript– please see line 609 (“However, the AAPI empathy construct had poor internal consistency for the current sample , whilst participants were likely already scoring at ceiling for this measure (scoring average of 46 out of a maximum of 50) and therefore it is difficult to draw strong conclusions as to the effectiveness of the VR in increasing empathy”) and 616 (“Internal consistencies were also poor for the other constructs of the AAPI, therefore conclusions drawn are limited and in particular for Oppressing Power and Independence”)

Concerning the suggestion to include behavioural measures, we fully agree. As noted in the conclusions (lines 813–815), we acknowledge the need for future work to integrate behavioural data alongside self-report measures. We have now made this point even more explicit by more prominently highlighting the absence of an objective behavioural measure in the current study and recommending its inclusion in future research (line 675).

4. The statistical analyses are appropriate, with non-parametric tests used where assumptions were violated. The results are presented clearly, but the lack of significant findings for empathy changes is disappointing. The qualitative findings provide valuable insights into participants' experiences, but the quantitative results are less compelling. The interaction between attachment style and parenting changes is interesting but preliminary due to the small sample.

- We thank the reviewer for this comment and feel that this evaluation is represented well in our manuscript already. We agree with the preliminary nature of the findings and mentioned earlier we have tried to highlight this throughout the manuscript.

5. The discussion acknowledges the study's limitations, such as the small sample and measure reliability issues. It contrasts the findings with previous research well but could explore more why empathy scores did not change. The implications for VR interventions are discussed, but the section could benefit from more specific recommendations for future research, such as larger, more diverse samples or improved measures.

- We thank the reviewer for the above comment. We have added specific recommendations for future research, in line with the reviewers’ suggestions, emphasising the need for larger and more diverse samples as well as improved measures (please see lines 48 - 54, 751, 675)
- Regarding the question of why empathy scores did not change, we believe the discussion provides a thorough exploration of several plausible explanations. These include issues with the face validity and reliability of the AAPI (e.g. lines 609, 616), using the same versions of the AAPI at pre- and post-assessment (line 622, Hamilton-Giachritsis et al., 2018 paper used different versions as per the guidance for the measure) which was done due to concerns regarding face validity of items contributing to certain constructs and consistency of these items across versions), possible ceiling effects in empathy (line 610), and a limited sample size (line 612). We also note the lack of validation of the REMT in the present sample and the absence of established evidence supporting its sensitivity to change in empathy (line 805). We have also highlighted the cultural differences between the current sample (British) and that used in Hamilton-Giachritsis et al., 2018 (Spanish) as a possible reason for not replicating findings. In addition, we discuss possible challenges related to immersion, embodiment and believability within the VR paradigm for the specific sample (lines 575, 634). Together, these points outline a comprehensive set of hypotheses which will hopefully inform and motivate future research in this area.

6. Strengths: The mixed-methods approach, focus on attachment styles, and exploration of VR's potential are commendable. The qualitative data enriches the findings.

Thank you for the feedback

7- Weaknesses: The small, homogeneous sample, poor reliability of some measures, and lack of significant empathy changes weaken the study. The study could have included follow-up behavioral assessments to validate self-reported parenting changes.

We thank the reviewer for this comment and fully agree that these represent important weaknesses of the study. These issues are acknowledged in the manuscript, and we have further strengthened these sections to ensure that readers interpret these findings with the limitations of the study in mind.

We also agree that follow-up behavioural assessments would have offered an additional layer of validation for the self-reported parenting changes. While such measures were beyond the scope of the current study, we now explicitly emphasise this point in the revised manuscript and highlight it as an important direction for future research. We hope these clarifications help contextualise the findings appropriately.

Reviewer #2: The studys main flaw are formal errors. The inconsistent presentation of statistical parameters (for example some means and SDs are not in italics)

- We thank the reviewer for pointing this out, we have thoroughly reviewed the manuscript and all incidents of statistical parameters are now in italic font.

and terms (sometimes its "positive mother", at other times "Positive mother"),

- We have now reviewed the manuscript and positive and negative mother are written in non-italicised font consistently (except where used as a heading in a table).

makes at times difficult to focus on the content of the study. Please go through the whole text and check for inconsistencies and make sure every open bracket has a closing bracket as well.

- We have reviewed the entire manuscript and ensured all brackets have been closed.

Additionally numbers under 12 should be verbalized and not written as numbers, please check the Result section carefully.

- This has now been changed throughout the manuscript

Furthermore, the reference style is not used consistent. Sometimes, line 569, author name and year is presented, while the majority of the time the Vancouver Style is used. Since Plos One requires the Vancouver Style, other reference styles should be avoided.

- Author names and publication years are provided where it supports the structure of the sentence, but all citations now also include the corresponding Vancouver-style reference numbers..

In terms of content, it should be discussed in more details, why embodiment worked for some participants but not for other. Do they, for example, differ in demographic variables or previous experiences?

- We thank the reviewer for raising this important point. As noted in the revised manuscript, we have highlighted this for an area of future research to more thoroughly examine why embodiment may be experienced differently across participants, including the potential influence of demographic factors and prior experiences (line 755). Because this issue was not a primary focus of the present study, our analyses were exploratory in nature and were not designed to provide definitive explanations for these differences. Therefore, we are unable to offer interpretations beyond informed speculation.
- In line with the reviewer’s suggestion, we conducted additional post-hoc analyses exploring associations between embodiment ratings (for both the positive and negative conditions) and key demographic factors (including age, years of education, household income). These analyses provided no evidence of meaningful relationships for the vast majority of variables. However, we did observe a positive relationship between participant age and the question “the woman acted as if I was a child (harsh condition; *r* (19) = .58) and “I felt much younger than I actually am” (harsh condition; *r* (19) = .50, *ps* <.05). However, given the high number of performed correlation tests, these results would no longer be significant after adjustment for multiple comparisons. For this reason, and to avoid overstating tentative findings, we do not overemphasise this point in the manuscript but make reference to the correlation tables that can be found in S9 (in “S9 File. Correlation Tables_Revised”) and have strengthened the discussion to emphasize that future research should more robustly explore demographic factors that might shape embodiment and immersion as stated above.

What should be measured in following studies to understand this phenomenon.

Suggestions for further psychological variables that could be measured in future research to help understand factors that influence embodiment and immersion such as feelings of self-consciousness whilst in the lab environment have now been incorporated (lines 788).

- Overall the study deals with an interesting topic, however the small number of participants, the ceiling effects and the missing embodiment for some participants makes it hard to generalize the findings, which was stated appropriately by the others. I do miss some alternative explanations for the null findings, especially in relation to attachment style.

We thank the reviewer for the thoughtful comments. We agree that the small sample size, ceiling effects, and variability in embodiment limit the generalisability of the findings, and we have aimed to emphasise these constraints clearly throughout the manuscript. With respect to alternative explanations for the null findings, we believe we have provided several plausible interpretations regarding the absence of empathy change (see previous responses). However, it is challenging to further speculate on null findings specifically related to attachment style given the lack of main effect on empathy itself. Given this, we have been cautious not to overinterpret the attachment-related analyses. We also hope that the framing of our study as preliminary, together with the explicit discussion of its key methodological limitations, makes clear that the findings should not be generalised at this point.

6. PLOS authors have the option to publish the peer review history of their article ([what does this mean?](https://eur03.safelinks.protection.outlook.com/?url=https%3A%2F%2Ftrack.editorialmanager.com%2FCL0%2Fhttps%3A%252F%252Fjournals.plos.org%252Fplosone%252Fs%252Feditorial-and-peer-review-process%2523loc-peer-review-history%2F1%2F010f0198d148a938-c77dab24-6c50-48c9-a04c-c1b05754caba-000000%2FEV4sJbkQuyoGH4QHE6pb6wtX-5aCX3GYblVpTx4DeTk%3D225&data=05%7C02%7Cairdriej%40cardiff.ac.uk%7C55641e78413f4db7fe0508de11f94fc9%7Cbdb74b3095684856bdbf06759778fcbc%7C1%7C0%7C638967959938228920%7CUnknown%7CTWFpbGZsb3d8eyJFbXB0eU1hcGkiOnRydWUsIlYiOiIwLjAuMDAwMCIsIlAiOiJXaW4zMiIsIkFOIjoiTWFpbCIsIldUIjoyfQ%3D%3D%7C0%7C%7C%7C&sdata=JTdyS0ih5u%2F8EjMMllIueOkADKFC%2FiFChmYbSpCLKcY%3D&reserved=0)). If published, this will include your full peer review and any attached files.

If you choose “no”, your identity will remain anonymous but your review may still be made public.

**Do you want your identity to be public for this peer review?** For information about this choice, including consent withdrawal, please see our [Privacy Policy](https://eur03.safelinks.protection.outlook.com/?url=https%3A%2F%2Ftrack.editorialmanager.com%2FCL0%2Fhttps%3A%252F%252Fwww.plos.org%252Fprivacy-policy%2F1%2F010f0198d148a938-c77dab24-6c50-48c9-a04c-c1b05754caba-000000%2FiqaPWtgILwLsbTt4_ln3eQlGi-Bm4gzqJG6_wCu85Zk%3D225&data=05%7C02%7Cairdriej%40cardiff.ac.uk%7C55641e78413f4db7fe0508de11f94fc9%7Cbdb74b3095684856bdbf06759778fcbc%7C1%7C0%7C638967959938242689%7CUnknown%7CTWFpbGZsb3d8eyJFbXB0eU1hcGkiOnRydWUsIlYiOiIwLjAuMDAwMCIsIlAiOiJXaW4zMiIsIkFOIjoiTWFpbCIsIldUIjoyfQ%3D%3D%7C0%7C%7C%7C&sdata=rPng2j%2BgV64wde%2B%2FPEubJpZVp9jSYIdQj8XAbxL50wE%3D&reserved=0).

Reviewer #1: **Yes:** Elham Baghban Baghestan

Reviewer #2: No

In addition to the responses to the reviewers’ comments, we have made further minor changes throughout the manuscript:

- We replaced the term “harmful” with “dysfunctional” when referring to parenting practices as measured by the Parenting Scale to be consistent with the terminology used by the original authors of this scale.
- At Line 746 – we changed “effectiveness” to “impact”
- At Line 761 – we refined the wording to highlight that some participants perceived the positive mother’s facial expression as briefly intimidating when she initially came into the room
