# Supplementary material for: Exploring the moderating role of maternal attachment style on the impact of a virtual reality environment on parental empathy and style: A pilot study
Source: PLoS One. 2026 Aug 24;21(8):e0355199. doi: 10.1371/journal.pone.0355199 (PMC13502691; doi:10.1371/journal.pone.0355199)
Supplement: Peer review history

# Peer review: Decision Letter 0

**DOI:** 10.1371/journal.pone.0355199.r001

**Authors:** Sara Ventura

22 Aug 2025

Dear Dr. Airdrie,

Thank you for submitting your manuscript to PLOS ONE. After careful consideration, we feel that it has merit but does not fully meet PLOS ONE’s publication criteria as it currently stands. Therefore, we invite you to submit a revised version of the manuscript that addresses the points raised during the review process.

Please submit your revised manuscript by Oct 04 2025 11:59PM. If you will need more time than this to complete your revisions, please reply to this message or contact the journal office at plosone@plos.org. When you're ready to submit your revision, log on to https://www.editorialmanager.com/pone/ and select the 'Submissions Needing Revision' folder to locate your manuscript file.

- A rebuttal letter that responds to each point raised by the academic editor and reviewer(s). You should upload this letter as a separate file labeled 'Response to Reviewers'.
- A marked-up copy of your manuscript that highlights changes made to the original version. You should upload this as a separate file labeled 'Revised Manuscript with Track Changes'.
- An unmarked version of your revised paper without tracked changes. You should upload this as a separate file labeled 'Manuscript'.

If you would like to make changes to your financial disclosure, please include your updated statement in your cover letter. Guidelines for resubmitting your figure files are available below the reviewer comments at the end of this letter.

If applicable, we recommend that you deposit your laboratory protocols in protocols.io to enhance the reproducibility of your results. Protocols.io assigns your protocol its own identifier (DOI) so that it can be cited independently in the future. For instructions see: https://journals.plos.org/plosone/s/submission-guidelines#loc-laboratory-protocols. Additionally, PLOS ONE offers an option for publishing peer-reviewed Lab Protocol articles, which describe protocols hosted on protocols.io. Read more information on sharing protocols at https://plos.org/protocols?utm_medium=editorial-email&utm_source=authorletters&utm_campaign=protocols.

We look forward to receiving your revised manuscript.

Kind regards,

Sara Ventura

Academic Editor

PLOS ONE

Journal Requirements:

When submitting your revision, we need you to address these additional requirements.

1. Please ensure that your manuscript meets PLOS ONE's style requirements, including those for file naming. The PLOS ONE style templates can be found at

https://journals.plos.org/plosone/s/file?id=wjVg/PLOSOne_formatting_sample_main_body.pdf and

https://journals.plos.org/plosone/s/file?id=ba62/PLOSOne_formatting_sample_title_authors_affiliations.pdf

2. Thank you for stating the following in the Competing Interests section:

“I have read the journal's policy and the authors of this manuscript have the following competing interests:

Domna Banakou (co-author) is an Academic Editor for Plos One

The authors have no other competing interests to declare.”

Please confirm that this does not alter your adherence to all PLOS ONE policies on sharing data and materials, by including the following statement: "This does not alter our adherence to PLOS ONE policies on sharing data and materials.” (as detailed online in our guide for authors http://journals.plos.org/plosone/s/competing-interests). If there are restrictions on sharing of data and/or materials, please state these. Please note that we cannot proceed with consideration of your article until this information has been declared.

Please include your updated Competing Interests statement in your cover letter; we will change the online submission form on your behalf.

3. If the reviewer comments include a recommendation to cite specific previously published works, please review and evaluate these publications to determine whether they are relevant and should be cited. There is no requirement to cite these works unless the editor has indicated otherwise.

Additional Editor Comments:

The article addresses an interesting topic. I have followed the authors’ work since the virtual environment was among the first adopted to study perspective-taking in VR with the paradigm of virtual embodiment. Applying the current VE to the study of attachment is ground-breaking. Although the clinical aspects of attachment are more complex and involve a broader range of emotional and psychological factors than empathy and perspective-taking alone, VR has shown potential as a tool to enhance these aspects and to address the issue of child maltreatment. However, the sample size is too small to allow for generalization of the results, although it could serve as a valuable pilot study. I recommend that the authors address the reviewers’ requirements before the article can be considered for publication.

[Note: HTML markup is below. Please do not edit.]

Reviewers' comments:

Reviewer's Responses to Questions

**Comments to the Author**

1. Is the manuscript technically sound, and do the data support the conclusions?

Reviewer #1: Partly

Reviewer #2: Yes

**********

2. Has the statistical analysis been performed appropriately and rigorously?

Reviewer #1: Yes

Reviewer #2: Yes

**********

3. Have the authors made all data underlying the findings in their manuscript fully available?

The PLOS Data policy

Reviewer #1: Yes

Reviewer #2: No

**********

4. Is the manuscript presented in an intelligible fashion and written in standard English?

Reviewer #1: Yes

Reviewer #2: No

**********

Reviewer #1: Dear Authors

congratulations on the work you have done. I read it throughly and personally I enjoyed a lot and honestly, it enlighted many lamps in my mind!

However, in order to enhance the quality of your manuscript I add some comments you may find useful:

1- The abstract provides a clear overview of the study's objectives, methods, and key findings. It mentions the sample size, design, and primary outcomes, such as the lack of change in empathy scores and the improvement in parenting attitudes. However, it could briefly highlight the qualitative findings, which add depth to the quantitative results. The abstract is concise but might benefit from a sentence summarizing the implications for future research or practice.

2. The study addresses an important topic—using VR to enhance parental empathy and reduce child maltreatment risk. The innovation lies in exploring how attachment styles moderate VR's effects. However, the study does not replicate previous findings on empathy changes, which limits its novelty. The focus on attachment styles adds value, but the small sample and homogeneity of participants reduce the generalizability of the results.

3. The within-groups, counterbalanced design is appropriate for testing VR's effects. The use of both quantitative and qualitative methods strengthens the study. However, the small sample size (N=19) and lack of diversity (high SES, highly educated) are major limitations. The measures, such as the AAPI, had poor reliability for some constructs, which affects the validity of the findings. The study could have included behavioral measures to complement self-reports.

4. The statistical analyses are appropriate, with non-parametric tests used where assumptions were violated. The results are presented clearly, but the lack of significant findings for empathy changes is disappointing. The qualitative findings provide valuable insights into participants' experiences, but the quantitative results are less compelling. The interaction between attachment style and parenting changes is interesting but preliminary due to the small sample.

5. The discussion acknowledges the study's limitations, such as the small sample and measure reliability issues. It contrasts the findings with previous research well but could explore more why empathy scores did not change. The implications for VR interventions are discussed, but the section could benefit from more specific recommendations for future research, such as larger, more diverse samples or improved measures.

6. Strengths: The mixed-methods approach, focus on attachment styles, and exploration of VR's potential are commendable. The qualitative data enriches the findings.

7- Weaknesses: The small, homogeneous sample, poor reliability of some measures, and lack of significant empathy changes weaken the study. The study could have included follow-up behavioral assessments to validate self-reported parenting changes.

wish you the best.

Reviewer #2: The studys main flaw are formal errors. The inconsistent presentation of statistical parameters (for example some means and SDs are not in italics) and terms (sometimes its "positive mother", at other times "Positive mother"), makes at times difficult to focus on the content of the study. Please go through the whole text and check for inconsistencies and make sure every open bracket has a closing bracket as well. Additionally numbers under 12 should be verbalized and not written as numbers, please check the Result section carefully. Furthermore, the reference style is not used consistent. Sometimes, line 569, author name and year is presented, while the majority of the time the Vancouver Style is used. Since Plos One requires the Vancouver Style, other reference styles should be avoided.

In terms of content, it should be discussed in more details, why embodiment worked for some participants but not for other. Do they, for example, differ in demographic variables or previous experiences? What should be measured in following studies to understand this phenomenon.

Overall the study deals with an interesting topic, however the small number of participants, the ceiling effects and the missing embodiment for some participants makes it hard to generalize the findings, which was stated appropriately by the others. I do miss some alternative explanations for the null findings, especially in relation to attachment style.

**********

what does this mean?). If published, this will include your full peer review and any attached files.

If you choose “no”, your identity will remain anonymous but your review may still be made public.

**Do you want your identity to be public for this peer review?**  For information about this choice, including consent withdrawal, please see our Privacy Policy

Reviewer #1: **Yes:** Elham Baghban Baghestan

Reviewer #2: No

**********

[NOTE: If reviewer comments were submitted as an attachment file, they will be attached to this email and accessible via the submission site. Please log into your account, locate the manuscript record, and check for the action link "View Attachments". If this link does not appear, there are no attachment files.]

While revising your submission, please upload your figure files to the Preflight Analysis and Conversion Engine (PACE) digital diagnostic tool, https://pacev2.apexcovantage.com/. PACE helps ensure that figures meet PLOS requirements. To use PACE, you must first register as a user. Registration is free. Then, login and navigate to the UPLOAD tab, where you will find detailed instructions on how to use the tool. If you encounter any issues or have any questions when using PACE, please email PLOS at figures@plos.org. Please note that Supporting Information files do not need this step.

# Author comment: Author response to Decision Letter 1

**DOI:** 10.1371/journal.pone.0355199.r002

8 May 2026

Please see attached "Response to reviewers v1.0 20 02 26" file for a full response to the reviewer's comments. In addition I can confirm that whilst Domna Banakou is an editor for PlosOne this does not alter our adherence to PLOS ONE policies on sharing data and materials.

Thank you.

Jac Airdrie

Attachment Submitted filename: Response to reviewers v1.0 20 02 26.docx

# Peer review: Decision Letter 1

**DOI:** 10.1371/journal.pone.0355199.r003

**Authors:** Laura Hannah Kelly

20 Jul 2026

Exploring the Moderating Role of Maternal Attachment Style on the Impact of a Virtual Reality Environment on Parental Empathy and Style: A Pilot Study

PONE-D-25-18141R1

Dear Dr. Airdrie,

We’re pleased to inform you that your manuscript has been judged scientifically suitable for publication and will be formally accepted for publication once it meets all outstanding technical requirements.

Within one week, you’ll receive an e-mail detailing the required amendments. When these have been addressed, you’ll receive a formal acceptance letter and your manuscript will be scheduled for publication.

An invoice will be generated when your article is formally accepted. Please note, if your institution has a publishing partnership with PLOS and your article meets the relevant criteria, all or part of your publication costs will be covered. Please make sure your user information is up-to-date by logging into Editorial Manager at Editorial Manager® and clicking the ‘Update My Information' link at the top of the page. For questions related to billing, please contact billing support.

If your institution or institutions have a press office, please notify them about your upcoming paper to help maximize its impact. If they’ll be preparing press materials, please inform our press team as soon as possible -- no later than 48 hours after receiving the formal acceptance. Your manuscript will remain under strict press embargo until 2 pm Eastern Time on the date of publication. For more information, please contact onepress@plos.org.

Kind regards,

Laura Kelly, PhD

Division Editor

PLOS One

Additional Editor Comments (optional):

Reviewers' comments:

Reviewer's Responses to Questions

**Comments to the Author**

Reviewer #1: All comments have been addressed

**********

2. Is the manuscript technically sound, and do the data support the conclusions?

Reviewer #1: Yes

**********

3. Has the statistical analysis been performed appropriately and rigorously?

Reviewer #1: Yes

**********

4. Have the authors made all data underlying the findings in their manuscript fully available?

The PLOS Data policy

Reviewer #1: Yes

**********

5. Is the manuscript presented in an intelligible fashion and written in standard English?

Reviewer #1: Yes

**********

Reviewer #1: Dear authors, thanks for your effrts for addressing my comments. wish you all the best in other research works.

**********

what does this mean?). If published, this will include your full peer review and any attached files.

If you choose “no”, your identity will remain anonymous but your review may still be made public.

**Do you want your identity to be public for this peer review?**  For information about this choice, including consent withdrawal, please see our Privacy Policy

Reviewer #1: **Yes:** Elham Baghban Baghestan

**********

# Editor report: Acceptance letter

**DOI:** 10.1371/journal.pone.0355199.r004

**Authors:** Laura Hannah Kelly

PONE-D-25-18141R1

PLOS One

Dear Dr. Airdrie,

I'm pleased to inform you that your manuscript has been deemed suitable for publication in PLOS One. Congratulations! Your manuscript is now being handed over to our production team.

At this stage, our production department will prepare your paper for publication. This includes ensuring the following:

* All references, tables, and figures are properly cited

* All relevant supporting information is included in the manuscript submission,

* There are no issues that prevent the paper from being properly typeset

You will receive further instructions from the production team, including instructions on how to review your proof when it is ready. Please keep in mind that we are working through a large volume of accepted articles, so please give us a few days to review your paper and let you know the next and final steps.

Lastly, if your institution or institutions have a press office, please let them know about your upcoming paper now to help maximize its impact. If they'll be preparing press materials, please inform our press team within the next 48 hours. Your manuscript will remain under strict press embargo until 2 pm Eastern Time on the date of publication. For more information, please contact onepress@plos.org.

You will receive an invoice from PLOS for your publication fee after your manuscript has reached the completed accept phase. If you receive an email requesting payment before acceptance or for any other service, this may be a phishing scheme. Learn how to identify phishing emails and protect your accounts at https://explore.plos.org/phishing.

If we can help with anything else, please email us at customercare@plos.org.

Thank you for submitting your work to PLOS One and supporting open access.

Kind regards,

PLOS ONE Editorial Office Staff

on behalf of

Dr. Laura Hannah Kelly

Staff Editor

PLOS One
